# Unveiling Vibrational Couplings in Model Peptides in Solution by a Theoretical Approach

**DOI:** 10.3390/molecules30132854

**Published:** 2025-07-04

**Authors:** Federico Coppola, Fulvio Perrella, Alessio Petrone, Greta Donati, Luciana Marinelli, Nadia Rega

**Affiliations:** 1 Scuola Superiore Meridionale, Largo San Marcellino 10, I-80138 Napoli, Italy; f.coppola@ssmeridionale.it (F.C.); f.perrella@ssmeridionale.it (F.P.); alessio.petrone@unina.it (A.P.); 2 Department of Chemical Sciences, University of Napoli Federico II, Complesso Universitario di M.S. Angelo, Via Cintia 21, I-80126 Napoli, Italy; 3 Istituto Nazionale di Fisica Nucleare, Sezione di Napoli, Complesso Universitario di M.S. Angelo, Via Cintia 21, I-80126 Napoli, Italy; 4 Department of Pharmacy, University of Napoli Federico II, Via Domenico Montesano 49, I-80131 Napoli, Italy; luciana.marinelli@unina.it

**Keywords:** ab initio molecular dynamics, DFT, model peptides, hydrogen bonding in aqueous solution, solvent effects on vibrational dynamics, environmental effects

## Abstract

Vibrational analysis of peptides in solution and the theoretical determination of the effects of the microenvironment on infrared and Raman spectra are of key importance in many fields of chemical interest. In this work, we present a computational study combining static quantum mechanical calculations with ab initio molecular dynamics simulations to investigate the vibrational behavior of three peptide models in both the gas phase and in explicit water, under non-periodic boundary conditions. The vibrational spectra of the main amide bands, namely amide I-III and A, were analyzed using a time–frequency approach based on the wavelet transform, which allows the resolution of transient frequency shifts and mode couplings along the trajectories. This combined approach enabled us to perform a time-resolved vibrational analysis revealing how vibrational frequencies, especially of the C=O and N–H stretching modes, evolve over time due to dynamical microsolvation. These fluctuations modulate vibrational couplings and lead to spectral broadening and frequency shifts that correlate with the local structuring of the solvent. In conclusion, our results highlight how the proposed protocol allows for the direct connection between vibrational modes and local structural changes, providing a link from the spectroscopic observable to the structure, the peptide backbone, and its microenvironment.

## 1. Introduction

Understanding the relationship between structure and function in complex molecular systems is a fundamental challenge in chemical sciences, with profound implications for a wide range of applications—from rational drug design [1,2,3,4] to the development of novel biomimetic materials and processes [5,6,7,8,9] Elucidating the mechanisms underlying molecular recognition, folding, and conformational dynamics requires experimental techniques capable of probing biomolecules in their native, often heterogeneous and complex chemical environments.

In this context, infrared (IR) and Raman spectroscopies [10,11,12], often complemented by chiroptical and magnetic resonance techniques such as vibrational circular dichroism and nuclear magnetic resonance [13,14,15,16,17] are powerful tools to characterize the conformational landscape of peptides, proteins, and nucleic acids [18]. These techniques are non-destructive, highly sensitive, and can be applied to systems in solution or in membranes, making them ideally suited for studying biomolecular structure under physiological conditions.

Techniques such as femtosecond-stimulated Raman spectroscopy (FSRS) [19,20,21,22] and two-dimensional infrared (2DIR) spectroscopy [23,24,25,26,27,28,29,30,31] can provide additional insights into the vibrational dynamics of biomolecules. The vibrational spectra of biomolecules contain detailed fingerprints of their secondary structure and interactions, such as band positions, intensities, and line shapes are modulated by hydrogen bonding, solvent polarity, and conformational flexibility. However, the correct interpretation of these spectral features is often non-trivial, requiring the support of theoretical and computational approaches in order to disentangle the underlying structural and dynamical contributions [32,33,34,35,36]. Peptides and proteins possess several IR-active vibrational modes, among which the amide bands are the most significant due to their sensitivity to the local environment, particularly through their sensitivity to hydrogen bonding [24,31,37,38,39,40,41,42]. The amide I band, centered around 1700 cm^−1^, originates primarily from the C=O stretching vibration and is highly responsive to hydrogen bond formation. In the presence of hydrogen bond donors—especially water—this band typically undergoes a red shift to lower frequencies, reflecting the weakening of the carbonyl bond due to electron delocalization. In aqueous environments, the amide I mode can also couple with the bending vibrations of water, leading to spectral broadening and complexity, but also offering additional information about solute– solvent interactions. The amide II band, near 1500 cm^−1^, arises from a combination of in- plane N–H bending and C–N stretching. In condensed phases, it often exhibits a blue shift, which can be attributed, in part, to the resonance structure of the secondary amide, which becomes more pronounced in the presence of hydrogen bonding. When the C=O and N–H groups are involved in hydrogen bonds—especially in aqueous solution—the electron delocalization across the amide bond increases. As a result, the C–N bond acquires partial double-bond character, leading to a shortening and stiffening of this bond. The amide III band, located around 1300 cm^−1^, is dominated by C–N stretching character and, while less directly linked to hydrogen bonding, still shows sensitivity to the surrounding molecular context. The behavior of the amide II and III modes provides important spectroscopic evidence for the subtle electronic and structural rearrangements induced by hydrogen bonding in polar environments. Finally, the amide A band, found near 3500 cm^−1^, corresponds to N–H stretching and is fully localized, making it a direct probe of hydrogen bond strength and directionality. In the presence of strong hydrogen bonding, this band shows a pronounced red shift, as the N–H bond becomes elongated due to its interaction with the oxygen atom [43,44,45,46,47,48,49]. While vibrational spectroscopy offers experimental access to structural information, theoretical approaches are essential to interpret, rationalize, and predict spectral features. Modern quantum chemical methods, particularly those based on density functional theory (DFT), provide a robust framework for calculating vibrational frequencies and intensities. DFT, in its hybrid version, has been vastly used for the theoretical characterization of both vibrational and dynamical properties of molecules and the description of the electronic structure in macromolecular systems of material or biological interest [50,51,52,53,54,55,56,57,58]. In the standard approach, vibrational modes are obtained from the diagonalization of the Hessian matrix—constructed from the second derivatives of the potential energy with respect to nuclear displacements—at the equilibrium geometry of the system [59]. However, this harmonic approximation neglects important features such as anharmonic couplings, finite-temperature effects, and solvent-induced fluctuations. To go beyond the harmonic treatment, perturbation theory to second order (PT2) is often employed, enabling the calculation of cubic and quartic force constants that account for anharmonicities and vibrational mode couplings [60,61,62]. While PT2 approaches are computationally efficient for small and medium-sized molecules, their application becomes impractical for large biomolecules or systems in condensed phase due to the high cost associated with Hessian calculations and the difficulty of limiting the analysis to minimum energy structures (that can be several for peptides and proteins). Furthermore, static methods inherently lack dynamical effects and cannot account for temporal fluctuations in vibrational features [63].

Ab initio molecular dynamics (AIMD) simulations [64,65,66,67] overcome these limitations by explicitly incorporating nuclear motion and sampling vibrational behavior at finite temperature, by also inherently sampling multiple regions of the potential accessible to the systems. Within this framework, vibrational spectra can be reconstructed from time-correlation functions of atomic velocities or dipole moments using the Fourier transform (FT), providing access to anharmonic and time-independent vibrational features. The concept of generalized normal modes [63,68,69], enables vibrational mode analysis along the AIMD trajectories to obtain detailed vibrational information beyond the static harmonic picture. However, a key limitation of the FT-based analysis is the loss of temporal localization. All frequency information is distributed globally over the trajectory, not allowing the monitoring of frequency shifts, transient interactions, or dynamical couplings as they evolve in time. To address this, we propose the use of the wavelet transform (WT) [70,71], a powerful mathematical tool that retains both frequency and time information. Unlike FT, WT enables the detection of frequency modulations on short time scales and has to be chosen for analyzing non-stationary signals such as those arising from vibrational dynamics in complex environments because of the intrinsic time-dependent nature of these signals that cannot be caught by the Fourier transform, which is capable of providing only average frequency contents.

Wavelet-based methods have been widely used in engineering, biomedical imaging, signal processing, and financial time series analysis [72,73,74,75,76,77], nonetheless, in recent years, some of the authors have successfully applied wavelet-based approaches to a range of computational spectroscopic studies, including investigations of excited state proton transfer in fluorescent protein chromophores and dyes [78,79], vibrational relaxation in charge-transfer complexes [80,81,82], peptide models in aqueous solution [83], and photoinduced reaction mechanism [84]. In this study, we employ a computational protocol developed by some of the authors (see Ref. [83]) that combines AIMD simulations with wavelet-based vibrational analysis. The systems investigated include trans-N-Methyl-Acetamide (tNMA), its through-space dimer, and Methyl-2-Acetamidopropanoate (AcAlaOMe).

These model peptides have been the subject of previous experimental and theoretical studies [83,85,86,87,88] while still presenting open questions regarding the interplay of hydrogen bond, anharmonicity, and environmental effects. The tNMA dimer provides a minimal yet chemically relevant representation of intermolecular hydrogen bonding between peptide units, thus allowing us to reliably probe donor and acceptor interactions. This system is particularly useful for isolating the spectroscopic signatures/fingerprints associated with hydrogen bond formation—with water or other surrounding peptide units—such as frequency shifts and anharmonic couplings in the amide bands, serving as a benchmark for longer/complex peptide systems. On the other hand, AcAlaOMe introduces a structural complexity through the presence of an *α*-carbon side chain and an ester-capped C-terminus, which mimic the chemical motifs found in real peptides. This model captures the main aspects of local conformational flexibility (dihedral rotations as well as intramolecular hydrogen bond that can compete—or cooperate—with solvation effects). As such, AcAlaOMe as a model peptide enables us to investigate the fine interplay between backbone conformation, inter- and intramolecular hydrogen bonds, and how all these interactions may collectively affect the vibrational response of the amide moiety.

In the present work, AIMD simulations were carried out in both gas and aqueous phases, employing a hybrid quantum mechanics/molecular mechanics (QM/MM) scheme [89,90] and non-periodic boundary conditions (NPBC) [91] for solvation. The model strategy incorporates explicit water molecules in the first few solvation shells, embedded in a dielectric continuum that mimics the bulk solvent environment. Our results highlight that a wavelet-based vibrational analysis enables detailed resolution of mode-specific frequency shifts, broadening effects, and peptide–solvent interactions. Indeed, we unveiled the temporal evolution of the couplings with low-frequency vibrational bands of the amide modes. This intrinsic complexity of these model systems in water solution is demonstrated by the wavelet analysis since the Fourier transform is not capable of catching it. The combined use of molecular dynamics and wavelet-based vibrational analysis, as presented here, offers a versatile framework that can be extended to complex systems such as intrinsically disordered proteins [92,93,94] or membrane-bound peptides [95,96,97]. Furthermore, this approach provides a theoretical basis for the molecular interpretation of time-resolved spectroscopic experiments, allowing the identification of transient vibrational signatures associated with structural dynamics and specific molecular interactions.

## 2. Results and Discussion

**Figure 1:**
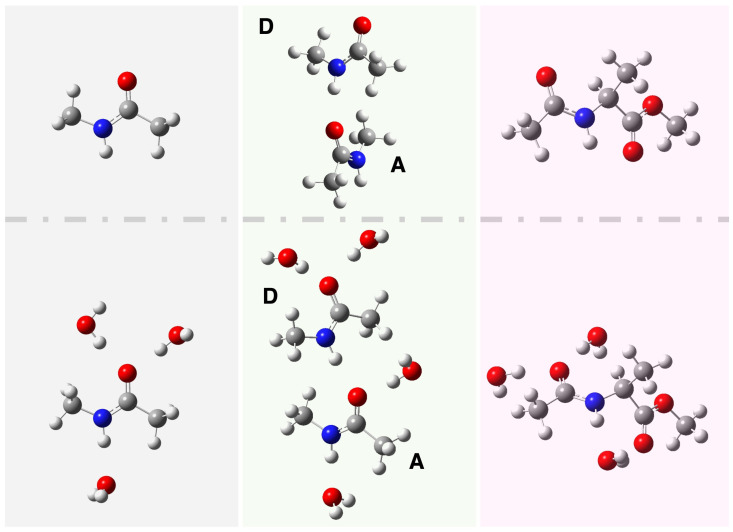
Molecular models investigated in this work. From left to right: trans-N-Methyl- Acetamide (tNMA), its hydrogen-bonded dimer (D: donor, A: acceptor), and Methyl-2-Acetamidopropanoate (AcAlaOMe). (**Top**): Optimized structures in C-PCM at the B3LYP/6-31G(d,p) level. (**Bottom**): Optimized solute–solvent cluster geometries in implicit solvent (C-PCM) at the same level of theory. Color code: carbon (gray), hydrogen (white), nitrogen (blue), oxygen (red).

We present here the results of our vibrational analysis performed on three peptide model systems: tNMA, its hydrogen-bonded dimer, and AcAlaOMe. Figure 1 schematically shows the investigated systems. In detail, the minimum energy structures without explicit water molecules (top panels) and where explicit water molecules saturate the primary solvation sites, such as N–H and C=O groups (bottom panels). In Figure S1, we report the peptide models optimized in the gas phase. By combining ab initio molecular dynamics (AIMD) simulations with a wavelet-based protocol, we tracked the evolution of vibrational frequencies over time, gaining qualitative insight into the couplings between vibrational modes. The computed vibrational features are compared to both anharmonic frequencies obtained via standard Hessian-based calculations and to available experimental data. In the following section, we present the structural characterization of the peptide models at finite temperature in solution and in the gas phase.

### 2.1. Structural Characterization in Gas and Aqueous Solution from AIMD Simulations

In Table 1, the main time-averaged structural parameters for the tNMA, its dimer, and AcAlaOMe are presented. The analysis from AIMD can catch the dynamical interactions between the solute and its aqueous environment lost by the static representation and can better explore conformational fluctuations. We observe systematic elongations of C=O and N–H bonds in the presence of water, reflecting strong hydrogen-bonding interactions with the solvent. This is accompanied by a decrease in the C=O bond order and a partial shift of the electronic density towards a zwitterionic resonance form. These findings are in agreement with previous studies on solvent effects in peptide systems [98]. In addition, a detailed structural characterization of the tNMA dimer and AcAlaOMe, based on the distributions of key structural parameters from both gas-phase and aqueous solution AIMD simulations are provided in Section S2.

**Figure 2:**
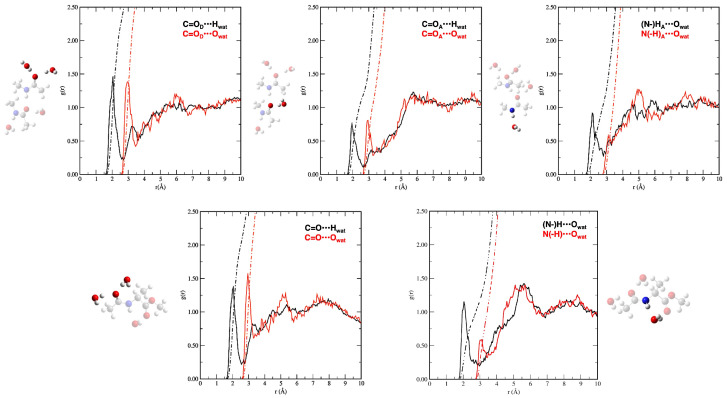
Pair radial distribution functions (RDFs) obtained from AIMD simulations in aqueous solution. (**Top panels**): RDFs for the tNMA dimer, with A and D indicating the monomers acting as acceptor and donor, respectively. (**Bottom panels**): RDFs for AcAlaOMe. Red lines represent the running coordination number (integrated RDF values). The corresponding molecule is shown faded on the side of the graph, with the relevant atom pair highlighted.

To gain deeper insight into microsolvation, we computed pair radial distribution functions (RDFs) from the AIMD trajectories. Figure 2 displays the RDFs for both the tNMA dimer and AcAlaOMe, focusing on interactions involving the amide groups: between carbonyl oxygens and water hydrogens (C=O⋯H_w_) and water oxygens (C=O⋯O_w_), as well as between amide hydrogens and water oxygens ((N)–H⋯O_w_) and amide nitrogens and water oxygens (N–(H)⋯O_w_). Integration of the RDFs yields the average number of water molecules participating in hydrogen bonds with each site. For the tNMA donor, a strong hydrogen bond is observed between the carbonyl oxygen and the surrounding water molecules. In detail, the C=O_*D*_⋯H_w_ RDF peaks at 2.05 Å with an integral of 2.31, indicating about two water molecules are hydrogen-bonded. The corresponding C=O_*D*_⋯O_w_ interaction, peaking at 2.95 Å with an integral of 2.50, further confirms significant solvation around the donor carbonyl group. In the case of the tNMA acceptor, the C=O_*A*_⋯H_w_ distance of 1.95 Å and an integral of 1.20 reveal weaker but still appreciable hydrogen bonding compared to the donor site. The C=O_*A*_⋯O_w_ interaction, peaking at 2.92 Å with an integral of 1.05, suggests a more limited hydration environment. The N–H hydrogen of the A monomer exhibits a single hydrogen-bonding interaction with water, characterized either by an (N)–H_*A*_⋯O_w_ distance peaking at 2.10 Å with an integral of 1.05, or by an N-(H)_*A*_⋯O_w_ distance peaking at 3.10 Å with an integral of 0.77. These distances highlight a moderately strong interaction with the solvent.

For AcAlaOMe, the RDFs show strong hydration at both the carbonyl and amide groups. The C=O⋯H_w_ interaction peaks at 2.10 Å with an integral of 2.27, while the corresponding C=O⋯O_w_ interaction occurs at 2.95 Å with an integral of 2.50, indicating extensive solvation of the carbonyl oxygen. The amide hydrogen exhibits an (N)–H⋯O_w_ interaction at 2.05 Å with an integral of 1.16, and the corresponding N-(H)⋯O_w_ interaction peaks at 3.05 Å with an integral of 0.98, consistent with efficient hydrogen bonding to water.

Overall, these results confirm the formation of well-defined hydration shells around both tNMA and AcAlaOMe, which, as discussed in the following sections, significantly influence their vibrational spectral features through hydrogen bonding. This analysis also drove the building of static cluster representations (in terms of water molecules to be included) and the protocol to extract the amide modes from the AIMDs.

### 2.2. Vibrational Analysis from AIMD Trajectories in Gas-Phase

**Figure 3:**
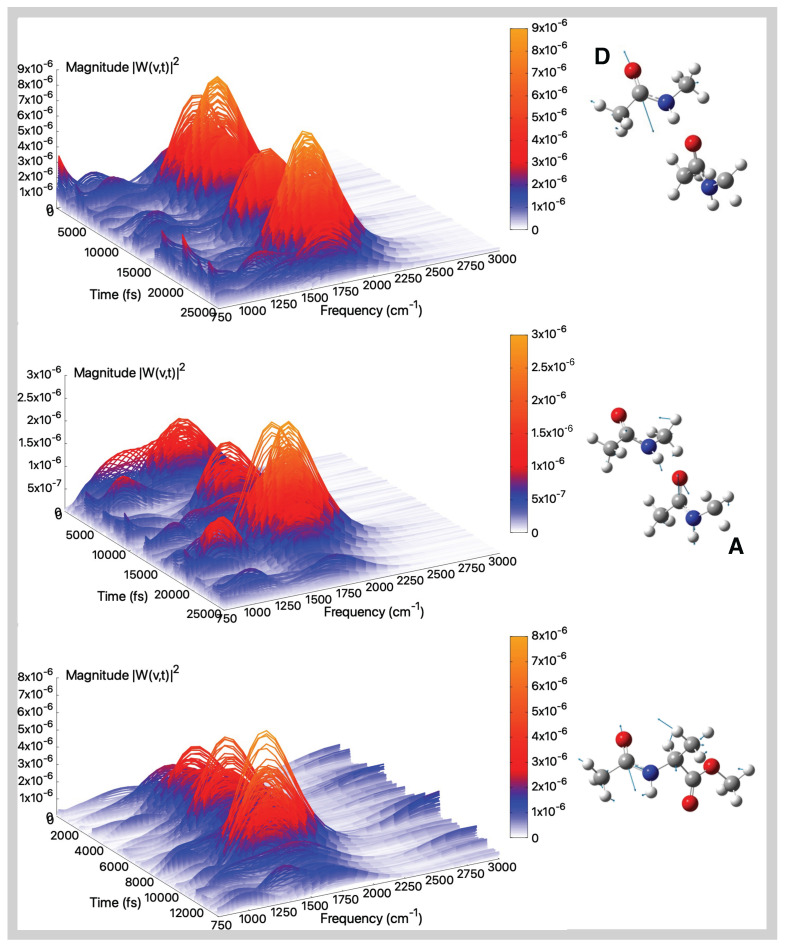
Amide I (gas-phase)—Time–frequency vibrational spectra obtained from the wavelet transform of velocity–velocity autocorrelation functions computed from AIMD simulations in the gas phase for the tNMA hydrogen-bonded dimer (Donor (D), Acceptor (A)), and AcAlaOMe systems, from top to bottom, respectively. The corresponding generalized normal mode compositions extracted from the trajectory are shown to the right of the spectrum. The *x*-axis represents time, the *y*-axis frequency (cm^−1^), and the intensity of the power spectrum is reported in the color scale (*z*-axis).

In this section, we provide a detailed characterization of the main vibrational modes (namely amide I, II, III, and A; see also Figure S6) as obtained from the analysis of gas-phase ab initio molecular dynamics trajectories. The analysis in the gas phase represents the starting point to understand and quantify the environmental effect on the amide modes. Indeed, it is known that these vibrational modes represent a fingerprint of peptides and proteins due to their high sensitivity to the environment. The Amide I mode of peptides serves as a sensitive marker for the secondary structure of polypeptides and proteins. It predominantly involves the stretching vibration of the carbonyl (C=O) group, resulting in an intense infrared absorption band (see Figure S6). Among the fundamental vibrational modes, Amide I is particularly responsive to environmental effects and is located in a spectral region largely free from overlapping bands. In Figure 3 (top and middle panels) are presented the amide I modes and corresponding wavelet spectra for the D and A monomers of the tNMA dimer system, obtained from the analysis of the AIMD trajectory in the gas phase. Regarding the D monomer (Figure 3, top panel), a well-resolved peak at approximately 1715 cm^−1^, with no significant contributions from other vibrational modes, is observed. Conversely, the amide I band of the A monomer (Figure 3, middle panel) is centered at 1698 cm^−1^. The carbonyl group of the A monomer is a hydrogen bond acceptor from the N–H group of the D monomer, triggering a red-shift of the band compared to the characteristic range in the gas phase, well-reproduced by the D monomer and in nice agreement with experimental values (1731 cm^−1^ [87]). In addition, the spectral features observed around 1100 cm^−1^ can be attributed, based on Hessian-based analysis, to bending vibrations of the methyl groups. This coupling, absent in the tNMA monomer (see Figure S8 in ESI), represents a characteristic feature of the through-space NMA dimer. Amide I of the D monomer also resembles the isolated tNMA peak, as shown by the Fourier and wavelet transforms presented in Figures S7 and S8. For both the monomer D and isolated tNMA, the wavelet spectra do not unveil additional information in terms of coupling or time-dependent behaviors compared to the Fourier transforms of the modes presented in Figures S7 and S9. A comparison of the wavelet spectra for the two monomers reveals that the A monomer shows additional lower-frequency contributions that are more significant compared to the D monomer (as also highlighted by the Fourier spectra in Figures S9 and S11). This behavior reflects the through-space interactions experienced by the C=O group of the A monomer, resulting in a more complex amide I mode whose spectral features are less straightforward to disentangle.

Finally, we consider the case of AcAlaOMe, an alanine-based analogue peptide (Figure 3, bottom panel). The amide I mode for this system is centered around 1708 cm^−1^, in agreement with previously reported theoretical calculations [99]. Similar to the A monomer of the tNMA dimer, the wavelet spectrum of AcAlaOMe displays non-negligible coupling with lower-frequency vibrational bands, although to a lesser extent and possibly due to the intrinsic, more complex nature of the molecule.

**Figure 4:**
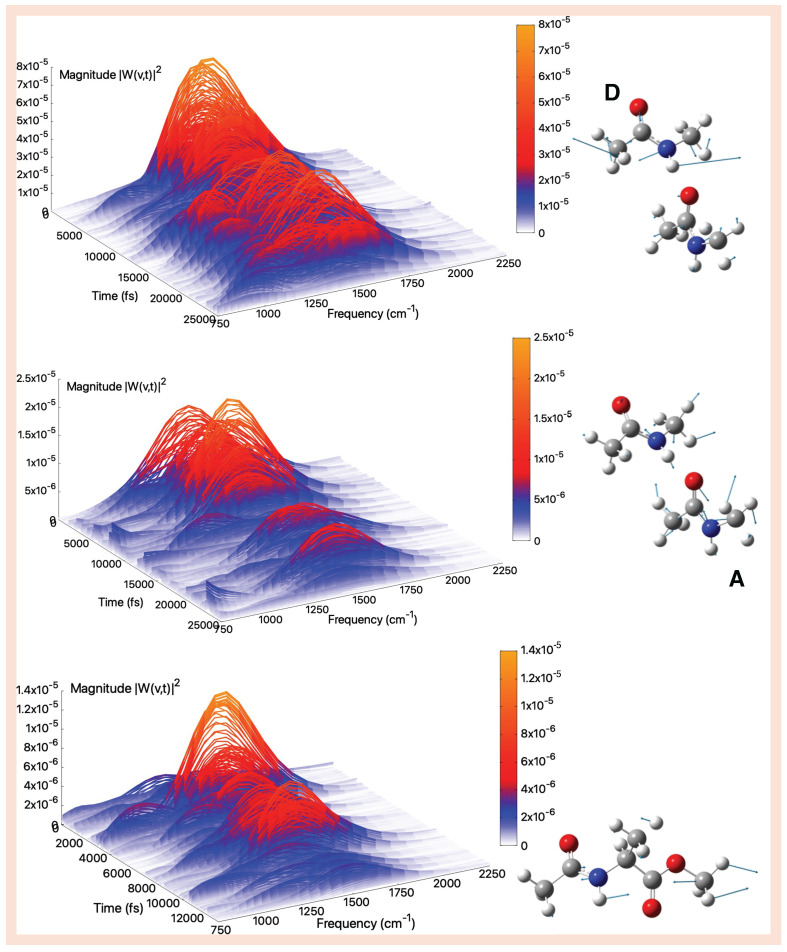
Amide II (gas-phase)—Time–frequency vibrational spectra obtained from the wavelet transform of velocity–velocity autocorrelation functions computed from AIMD simulations in the gas phase for the tNMA hydrogen-bonded dimer (Donor (D), Acceptor (A)), and AcAlaOMe systems, from top to bottom, respectively. The corresponding generalized normal mode compositions extracted from the trajectory are shown to the right of the spectrum. The *x*-axis represents time, the *y*-axis frequency (cm^−1^), and the intensity of the power spectrum is reported in the color scale (*z*-axis).

Figure 4 (top and middle panels) shows the amide II modes and spectra for the tNMA dimer. The amide II mode of peptides is typically observed in the 1510–1580 cm^−1^ spectral region and is more structurally complex than amide I. The mode primarily consists of an out-of-phase combination of N–H in-plane bending and partial C–N stretching motions (Figure S6). The presence of neighboring functional groups or solvation effects, particularly in aqueous environments, generally induces a blue-shift of approximately 80 cm^−1^ due to the concomitant decrease and increase of the C=O and C–N bond orders, respectively. In this case, the modes involve significant contributions from both monomers, resulting in a broader spectral distribution compared to the amide I band. As observed for the amide I modes, intermolecular interactions lead to frequency shifts relative to the isolated molecule. Indeed, in the monomer tNMA case (Figure S8), the maximum of the spectra is located at 1497 cm^−1^, in excellent agreement with the experimental value of 1499 cm^−1^ [87]. In the dimer case, the averaged maximum spectral values are found at 1515 cm^−1^ and 1505 cm^−1^ for the A and D monomers, respectively, thus confirming a notable blue-shift relative to the isolated tNMA. These gas-phase values can be compared to experimental observations in low-polarity solvents, where the amide II band of the tNMA dimer appears as a single broad feature centered at 1530 cm^−1^ in CCl_4_ [100] and at 1555 cm^−1^ in CH_2_Cl_2_ [101]. Notably, this vibrational mode overlaps with the spectral range associated with the scissoring motion of methyl groups, making its isolation and assignment particularly challenging.

Finally, Figure 4, bottom panel, reports the amide II wavelet spectrum for AcAlaOMe. The averaged spectral maximum is located at 1470 cm^−1^, which is reasonably consistent with that of isolated tNMA, given that the N–H group does not engage in significant electrostatic interactions with the surrounding environment. The band appears more complex, with multiple contributions arising from an incomplete separation of the mode. In all cases, the intrinsic complexity of the mode is also detected by the Fourier transforms (see Figures S7, S9, S11 and S13).

The amide III band arises from a combination of C–N stretching, N–H in-plane bending, and methyl group bending motions; see Figure S6. This band typically undergoes a blue-shift in aqueous environments due to specific interactions with surrounding water molecules.

**Figure 5:**
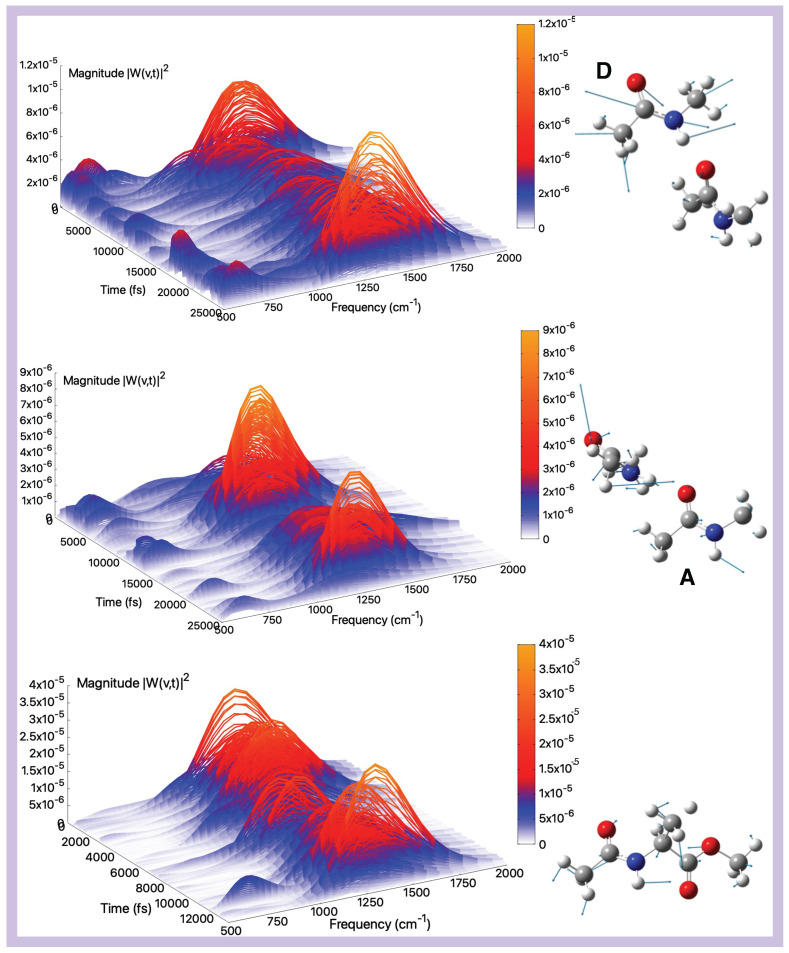
Amide III (gas-phase)—Time–frequency vibrational spectra obtained from the wavelet transform of velocity–velocity autocorrelation functions computed from AIMD simulations in the gas phase for the tNMA hydrogen-bonded dimer (Donor (D), Acceptor (A)), and AcAlaOMe systems, from top to bottom, respectively. The corresponding generalized normal mode compositions extracted from the trajectory are shown to the right of the spectrum. The *x*-axis represents time, the *y*-axis frequency (cm^−1^), and the intensity of the power spectrum is reported in the color scale (*z*-axis).

Figure 5 top and middle panels show the amide III modes and corresponding spectra for the tNMA dimer system, while Figure 5 reports the results for AcAlaOMe at the bottom panel. For the tNMA dimer case, the amide III frequencies are found at 1247 cm^−1^ and 1243 cm^−1^ for the A and D monomers, respectively, with the A monomer slightly blue-shifted compared to isolated tNMA (1244 cm^−1^, see Figure S8). This shift can be attributed to through-space interactions between the two monomers, leading to an increase in the C–N bond order and consequently a blue-shift in the A monomer’s amide III mode. For both the tNMA monomer and dimer, there are no other significant contributions in the same frequency range, especially in the first case, as also highlighted by the Fourier transforms of the mode (see Figures S7, S9, and S11).

Regarding AcAlaOMe, the amide III band appears red-shifted relative to tNMA and tNMA dimer, with a maximum located around 1215 cm^−1^. The wavelet spectra further reveal a coupling with methyl bending motions around 1380 cm^−1^. The latter feature can also be caught by the Fourier transform of the mode, displaying a wide vibrational band encompassing both modes (see Figure S13). However, in all cases there is a slight underestimation of the experimental value registered at around 1265 cm^−1^ [87].

The amide A mode is highly localized and primarily originates from a strong N–H stretching motion, appearing in the highest frequency region of the infrared spectrum (3300–3500 cm^−1^); see Figure S6. Its vibrational nature is highly anharmonic compared to other peptide fingerprint modes, and also many overtones and combination bands of lower frequency fall in this region [102].

**Figure 6:**
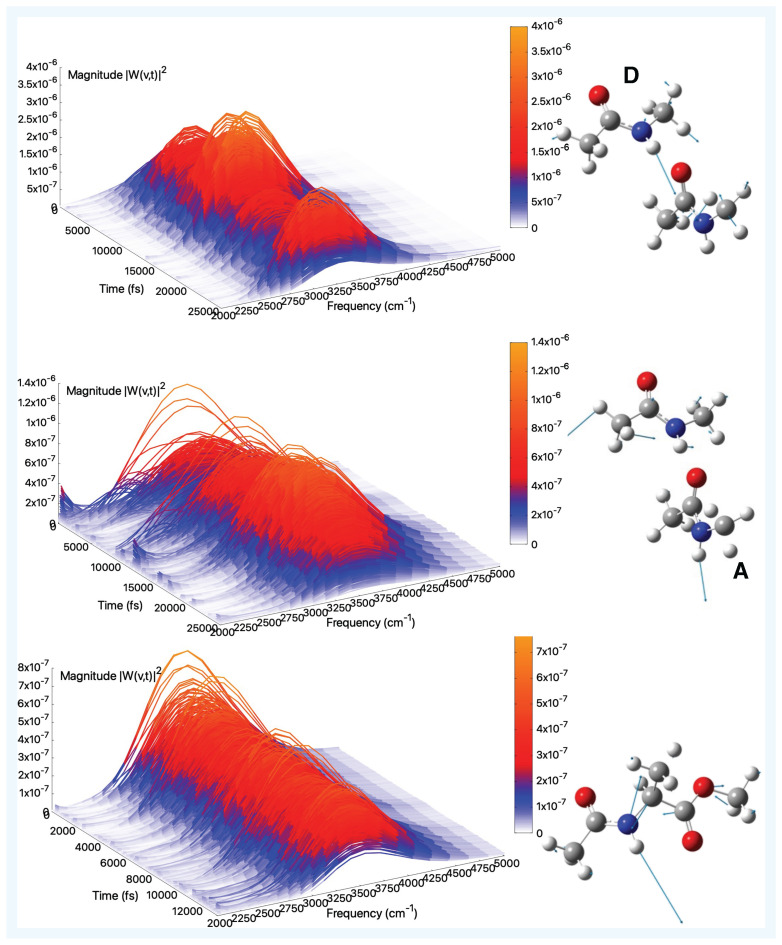
Amide A (gas-phase)—Time–frequency vibrational spectra obtained from the wavelet transform of velocity–velocity autocorrelation functions computed from AIMD simulations in the gas phase for the tNMA hydrogen-bonded dimer (Donor (D), Acceptor (A)), and AcAlaOMe systems, from top to bottom, respectively. The corresponding generalized normal mode compositions extracted from the trajectory are shown to the right of the spectrum. The *x*-axis represents time, the *y*-axis frequency (cm^−1^), and the intensity of the power spectrum is reported in the color scale (*z*-axis).

The tNMA dimer unveils two distinct amide A bands associated with the two monomers, as illustrated in the top and middle panels of Figure 6. In monomer A, where the N–H group is not significantly affected by intermolecular interactions, the amide A band is centered at 3533 cm^−1^, closely matching the IR peak observed at 3498 cm^−1^ in isolated matrices [103]. Conversely, in monomer D the amide A band corresponding to the hydrogen-bonded N–H stretching is strongly red-shifted and appears at 3167 cm^−1^. This large redshift not discussed in the literature could be a result of an overestimation of the hydrogen-bonding interactions sampled during the AIMD simulations.

The amide A mode extracted from the gas phase AIMD simulations of AcAlaOMe, together with the corresponding spectrum, is reported in Figure 6. This stretching motion remains highly localized, resulting in an intense band that dominates the high-frequency region of the infrared spectrum. The calculated peak position is 3508 cm^−1^, which compares reasonably well with the experimental value of 3452 cm^−1^ [23].

In the isolated tNMA molecule, the amide A mode, detected at 3511 cm^−1^, is a well-defined and isolated band with no other significant contribution (see Figures S7 and S8).

### 2.3. Vibrational Analysis from AIMD Trajectories in Aqueous Solution

In this section the wavelet analysis of the amide modes extracted from AIMD simulations in aqueous solution is presented. We calculated time-independent (from Fourier transform) and time-dependent (from wavelet transform) spectra of the peptide fingerprint bands (AI, AII, AIII, and AA) and analyzed the evolution in time of the band shapes, frequency shifts, and couplings with interacting water molecules modes. From the structural analysis, we noticed (see Figure 2) that, on average, two and one water molecules are hydrogen bonded with C=O and N–H groups, respectively. Therefore, we performed the vibrational analysis by considering velocities of the three molecules and of the water molecules engaging specific interactions with them. In this way we properly include the effect of the interaction with the solvent in the mode characterization.

**Figure 7:**
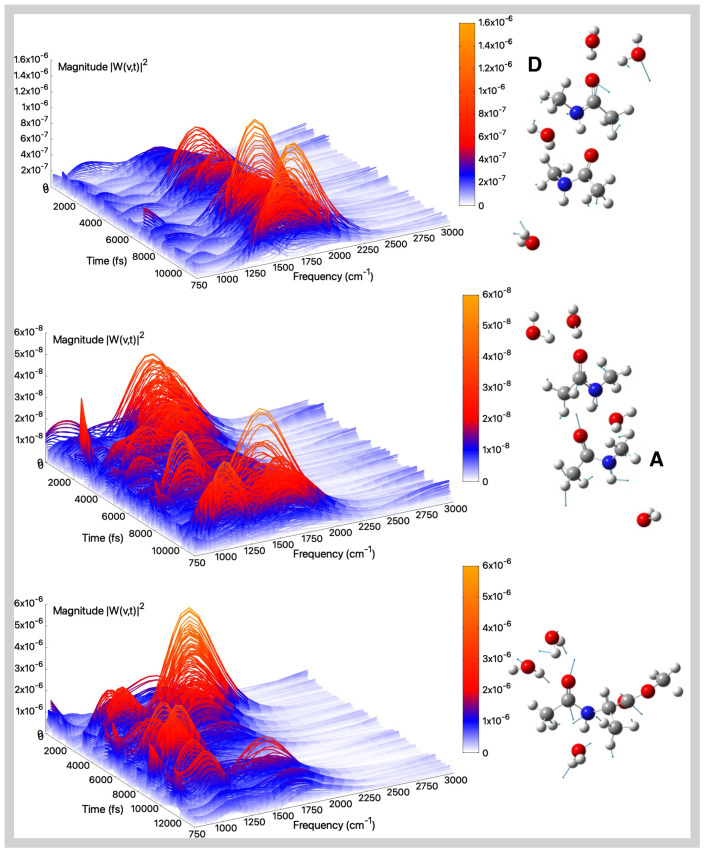
Amide I (aqueous solution)—Time–frequency vibrational spectra obtained from the wavelet transform of velocity–velocity autocorrelation functions computed from AIMD simulations in aqueous solution for the tNMA hydrogen-bonded dimer (Donor (D), Acceptor (A)), and AcAlaOMe systems, from top to bottom, respectively. The corresponding generalized normal mode compositions extracted from the trajectory are shown to the right of the spectrum. The *x*-axis represents time, the *y*-axis frequency (cm^−1^), and the intensity of the power spectrum is reported in the color scale (*z*-axis).

The amide I mode is the most sensitive for probing the environment, undergoing a significative red shift in aqueous solution of about 130 cm^−1^ for tNMA [87]) due to the interaction between the C=O group and the neighboring water molecules. The amide I modes and spectra obtained from the analysis of the tNMA dimer AIMD in aqueous solution are shown in Figure 7 top and middle panels. The bands show peaks centered at ca. 1655 and 1686 cm^−1^ for monomers A and D, respectively. We recall that the carbonyl group of the A monomer is hydrogen bonded to the monomer D N–H group and, on average, with one water molecule. In monomer D two water molecules interact on average with the C=O group, decreasing its strength. These interactions are responsible for the observed frequency red-shifts with respect to the gas phase (ca. −43 and −29 cm^−1^ for A and D monomers). These frequencies are instead blue-shifted with respect to the amide I of tNMA in aqueous solution reported in literature [83], showing a spectrum with a maximum value at 1620 cm^−1^ in excellent agreement with the observed one [87]. These differences indicate that the intermolecular interactions between monomers result in a lower weakening of the C=O bonds. The amide I band associated with the A monomer appears much more complex than the corresponding one computed in the gas phase. Indeed, beside the main peak centered at 1655 cm^−1^ we can observe a coupling with a minor band at slightly lower frequency values. In particular, such a spectral feature is around 1050 cm^−1^ and can be ascribed to an out-of-plane bending motion of the O=C-CH_3_ moiety, in line with the normal mode calculated by the Hessian-based approach at that frequency. As regards the amide I of the D monomer, a weak shoulder at 1677 cm^−1^ can also be observed, arising from the coupling with the bending of the surrounding water molecules. This vibrational feature is experimentally observed [104] and has been often related to two kinds of solute–solvent interaction. Concerning AcAlaOMe, as shown in Figure 7 bottom panel, we obtained more crowded spectra with respect to those calculated in the gas phase due to frequency fluctuations, couplings with water-bending motions, and ester carbonyl stretching. From a closer inspection of the wavelet spectrum, we identify a spectral contribution around 1100 cm^−1^. From the normal modes analysis (both static and from AIMD), suggest that this band can be assigned to deformations involving the later chain of *α*-carbon and the N-C*α* stretching. Furthermore, the amide I mode results are difficult to resolve because of the presence of methyl groups and a larger number of low-frequency modes activated. The observed peak is redshifted of ca. −43 cm^−1^ with respect to the isolated system and spans over a frequency range from 1680 to 1723 cm^−1^, which are frequency values associated to amide and ester carbonyl groups, respectively. However, the wavelet spectrum clearly shows that the major contribution still derives from the amide I mode. It is worth noting that the effects of all the surrounding waters in the generalized normal modes analysis are included. On the other hand, regarding the Hessian-based approach, we did not model a specific microsolvated cluster with water molecules hydrogen-bonded to the ester C=O group, since the AIMD simulations revealed a fluxional behavior in the solvent environment.

The Fourier transform of these modes (presented in Figures S10, S12 and S14 resembles these behaviors, although a clear picture of the coupling with other bands and their evolution during the time as observed thanks to the wavelet analysis cannot be caught.

**Figure 8:**
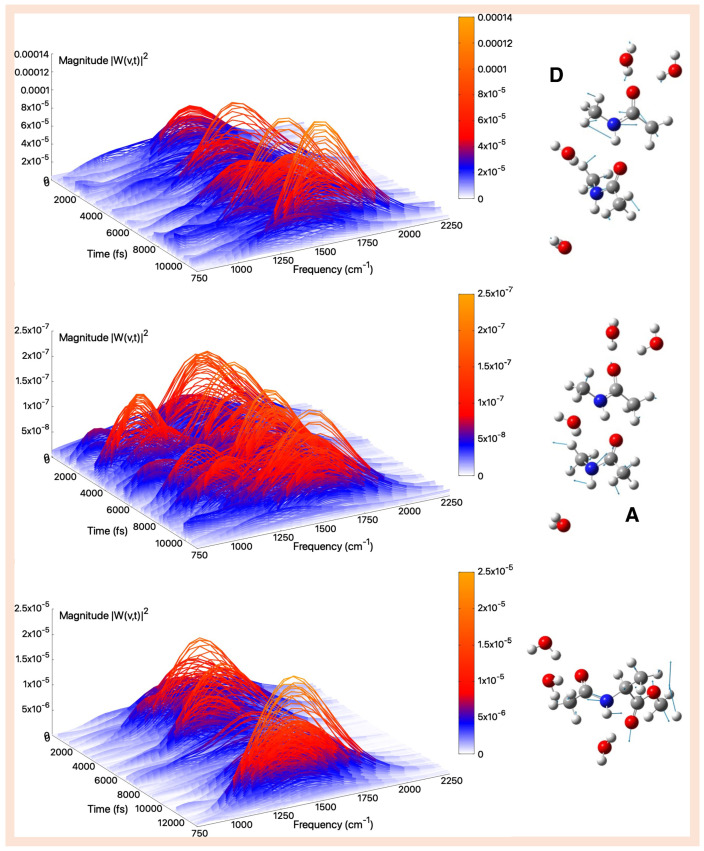
Amide II (aqueous solution)—Time–frequency vibrational spectra obtained from the wavelet transform of velocity–velocity autocorrelation functions computed from AIMD simulations in aqueous solution for the tNMA hydrogen-bonded dimer (Donor (D), Acceptor (A)), and AcAlaOMe systems, from top to bottom, respectively. The corresponding generalized normal mode compositions extracted from the trajectory are shown to the right of the spectrum. The *x*-axis represents time, the *y*-axis frequency (cm^−1^), and the intensity of the power spectrum is reported in the color scale (*z*-axis).

Among the fingerprint modes, the amide II band is often used for elucidation about secondary structure of proteins and polypeptides. In aqueous solution it exhibits a large blue shift with respect to the gas phase (ca. 80 cm^−1^) due to a larger sp^2^ character of the N-C bond. Recognition and assignment are not straightforward because of the presence of other active modes in the same spectral range, such as methyl rotations. The amide II modes and bands calculated for the tNMA dimer are shown in Figure 8 top and middle panels. Maximum values are centered at 1551 and 1549 cm^−1^ for monomers A and D, respectively. They are blue-shifted by 36 and 44 cm^−1^ with respect to the corresponding ones in the gas phase. The experimental trend is qualitatively reproduced, although a quantitative agreement is not completely achieved, possibly due to the MM representation of water molecules, where a single charge replaces the two lone pairs of electronic density of the water oxygen. Temporal evolution of interactions between hydrophilic sites and neighboring water molecules makes the spectra more crowded and complex, with respect to the gas phase. This is particularly evident for monomer A, where we observe vibrational modes around 1100 cm^−1^ (N-CH_3_ stretching) and around 1050 cm^−1^ (the out-of-plane bending involving the acetyl group). For monomer D, the contributions are less pronounced; we observe signals in the 1200–1300 cm^−1^ indicating couplings with methyl group deformations and with amide III. From the vibrational analysis performed on AcAlaOMe in aqueous solution, the amide II band appears to be well-separated, with a frequency centered around 1510 cm^−1^, as the wavelet spectra points out in Figure 8 bottom panel. Additional contributions are found around 1200 cm^−1^, likely arising from methyl group motions, and spectral components around 1300 cm^−1^ may be attributed to coupling with the amide III mode, as also observed for the amide II band in tNMA monomer D. Electrostatic interactions with explicit water molecules alter the electronic structure of the amide bond, resulting in a blue shift of the frequency of ca. 41 cm^−1^ with respect to the isolated system. The quantitative reproduction of the typical amide II blue shift is challenging also in the tNMA monomer, as reported in literature [83]. Indeed, in the performed wavelet analysis, a large band centered around 1510 cm^−1^, i.e., blue-shifted of ca. 30^−1^ with respect to the analog value calculated in the gas phase, was found.

In a polar solvent such as water, the expected blue shift for the amide III is of about 60 cm^−1^ (referring to tNMA system). Among fundamental peptide bands, the amide III region is generally neglected due to its low signal. Since there is no water interference in the amide III region and, more importantly, the different secondary structures of proteins have more resolved differences in their amide III spectra, it is quite promising to use this latter to estimate protein secondary structure content [105]. Concerning the tNMA dimer, we found two distinct amide III bands placed around 1294 (monomer A). and 1281 cm^−1^ (monomer D), with a blue shift with respect to the gas phase of 47 and 38 cm^−1^ in the first and the second case, respectively. These results highlight the larger sensitivity of the acceptor to the solvent perturbation. Due to the intrinsic complex nature of the mode, the wavelet spectra associated with the amide III modes look crowded.

In the wavelet spectrum of monomer A, we observe bands around 1640 cm^−1^, likely due to water bending vibrations, as well as lower frequency collective modes at 860 and 600 cm^−1^, which are associated with methyl group motions and water librational modes, respectively. It is also interesting to highlight that for monomer D, the same group of modes discussed above is also present, but the appearance of a band around 1500 cm^−1^ suggests a coupling with the AII mode.

**Figure 9:**
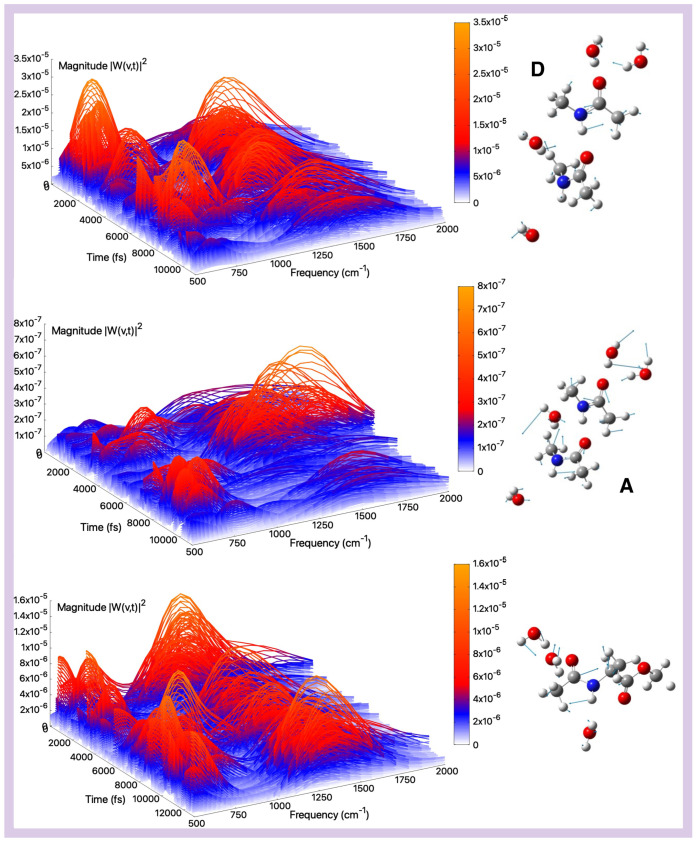
Amide III (aqueous solution)—Time–frequency vibrational spectra obtained from the wavelet transform of velocity–velocity autocorrelation functions computed from AIMD simulations in aqueous solution for the tNMA hydrogen-bonded dimer (Donor (D), Acceptor (A)), and AcAlaOMe systems, from top to bottom, respectively. The corresponding generalized normal mode compositions extracted from the trajectory are shown to the right of the spectrum. The *x*-axis represents time, the *y*-axis frequency (cm^−1^), and the intensity of the power spectrum is reported in the color scale (*z*-axis).

In the AcAlaOMe, the amide III mode appears at 1290 cm^−1^ (see Figure 9), ca. 75 cm^−1^ blue-shifted with respect to the corresponding ones extracted from AIMD simulation in the gas phase. Overall, in all cases the Fourier spectra appear less resolved compared to the corresponding ones in the gas phase (Figures S10, S12 and S14). Notably, the results of the dimer case resemble those of the tNMA monomer, where only a modest blue shift (about 23 cm^−1^) is observed relative to the gas-phase simulations [83]. This evidence suggests that the sensitivity of the amide III mode to the specific solute–solvent interactions is lower in the dimer compared to AcAlaOMe. Furthermore, the amide III mode appears to be less localized and more strongly coupled than other vibrational modes. In particular, the wavelet spectrum reveals the presence of multiple contributions: bands below 700 cm^−1^ associated with librational motions of interacting water molecules and bands around 1550 cm^−1^, indicating coupling with the AII mode, consistent with what was observed for the tNMA dimer (subunit D) and as extensively reported in the literature [88,106,107]. These complex features, and especially their time evolution, are unveiled thanks to the wavelet analysis, since from the Fourier transform the degree of coupling with the amide III mode could not be properly caught.

**Figure 10:**
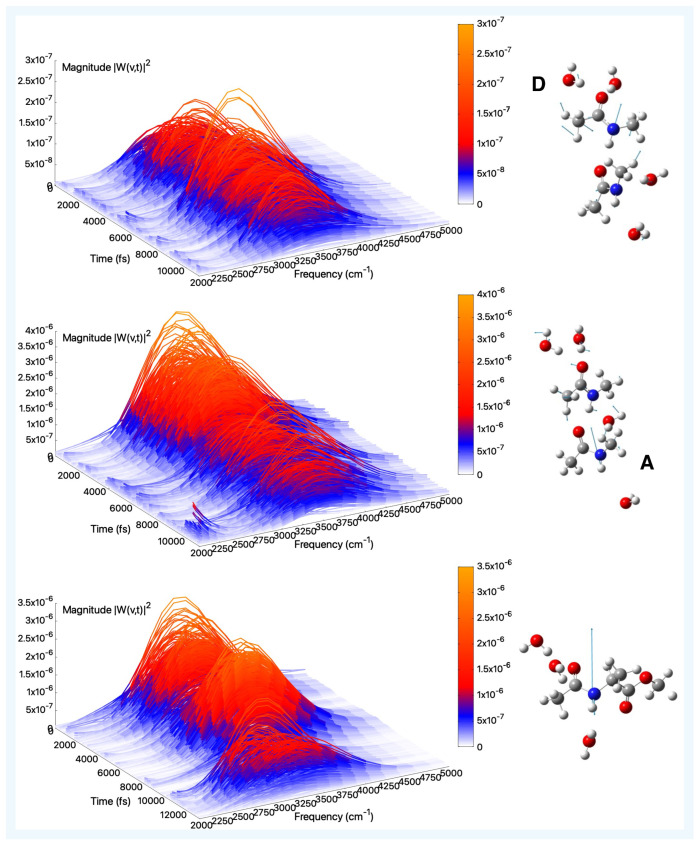
Amide A (aqueous solution)—Time–frequency vibrational spectra obtained from the wavelet transform of velocity–velocity autocorrelation functions computed from AIMD simulations in aqueous solution for the tNMA hydrogen-bonded dimer (Donor (D), Acceptor (A)), and AcAlaOMe systems, from top to bottom, respectively. The corresponding generalized normal mode compositions extracted from the trajectory are shown to the right of the spectrum. The *x*-axis represents time, the *y*-axis frequency (cm^−1^), and the intensity of the power spectrum is reported in the color scale (*z*-axis).

Finally, the amide A in aqueous solution is discussed. The N–H modes in aqueous solution are expected to shift toward lower frequencies by the formation of hydrogen bonds. From the analysis of the tNMA dimer shown in Figure 10 top and middle panels, we find two amide A bands that are very similar in their complex shapes, with a number of sub-bands. Both are weakly coupled with water O-H stretching. The spectra highlight the dynamical behavior of the monomer-monomer breathing (C=O—H-N) and monomer-water (N-H—OH_2_) interactions. Regarding the monomer A, we find a narrow peak centered at 3490 cm^−1^, the red-shift observed with respect to the corresponding one in the gas phase being of −45 cm^−1^. For the amide A of monomer D, we observe a double peak at 3423 and 3441 cm^−1^. In this case the amide A exhibits a considerable blue shift with respect to the analogous system in the gas phase, of 265 cm^−1^. However, this behavior is possibly due to the underestimated value recorded in the gas phase. A slightly red-shifted (−61 cm^−1^) amide A band is centered at 3447 cm^−1^ for the AcAlaOMe N–H group (see Figure 10). The band is not sharp as the same system in the gas phase, but it is more complex and covers a wider spectral range. This feature takes into account the extent of the various interactions with the surrounding solvent molecules.

### 2.4. Hessian-Based vs. AIMD-Based Vibrational Analysis

Here, we directly discuss the vibrational analysis of the amide modes by comparing the Hessian-based approach with the vibrational analysis from AIMD, and results are reported in Table 2 and Table 3. In Section S4, the Hessian-based vibrational analysis performed on the three model systems in gas and in condensed phases is reported for completeness. In Table 2 are presented the amide modes in the gas phase for the tNMA monomer, tNMA dimer, and AcAlaOMe systems. Regarding the amide I, the tNMA dimer and AcAlaOMe slightly overestimate the experimental values in both the static and dynamic pictures. Conversely, in the tNMA monomer case, the dynamic description underestimates the experimental frequency (1723 vs. 1731 cm^−1^). In all cases, the dynamic picture is the one more closely resembling the experimental values, with differences of maximum 17 cm^−1^ for the tNMA monomer and dimer and reaching larger discrepancy only in the AcAlaOMe case. These results suggest the capability of the AIMD to correctly and quantitatively simulate the vibrational features of these model systems. The trends achieved for AII and AIII are different for the static and dynamic pictures. Indeed, for the tNMA monomer, analysis from AIMD is still better compared to the static one (1497 vs. 1499 cm^−1^), while for the tNMA dimer, the trend is inverted. Both the intrinsic, more complex nature of the normal mode (involving simultaneously more contributions compared to the AI) and the interdependency of the monomers on each other in the dimer case impacting the nature of the mode contribute to a larger difficulty of an exhaustive description of the AII through the dynamic picture. A similar scenario is found for the AIII mode, although in this case also the tNMA monomer mode is better described by the Hessian-based representation. Regarding AcAlaOMe, there is a discrepancy of about 26 cm^−1^ between the AII static and dynamic descriptions and of 17 cm^−1^ for the AIII case. However, in these cases, there are no reference values from literature. Finally, for the amide A, both descriptions provide satisfactory agreement with the experimental data. However, in the dynamic representation, the difference between the two contributions of each tNMA monomer of the dimer is more emphasized. This feature could be ascribed to the methodology in terms of extraction of the normal modes from AIMD, possibly overestimating especially the red-shift of the D monomer. Overall, these results highlight that the analysis of generalized normal modes from AIMD is capable of accurately describing the nature of the amide modes and can be a valuable alternative to the Hessian-based approach.

In Table 3, the vibrational analysis comparison for the two approaches in aqueous solution is presented. First, the full-QM picture of the systems in implicit solvent is compared to the experimental values. It is evident that the implicit solvent picture fails in properly describing the effect of the environment. Indeed, for all three systems, amide I is computed at 1728, 1683/1702, and 1701 cm^−1^ for the tNMA monomer, tNMA dimer (A/D), and AcAlaOMe, respectively. The characteristic redshift of this mode is only qualitatively reproduced. Since the main reason for the redshift is represented by the specific interactions established with water molecules, the C-PCM picture is intrinsically incapable of catching it. Indeed, a more quantitative simulation of the red-shift is achieved by the cluster pictures in all cases. The latter resemble more closely the frequencies extracted from the AIMD that is capable of furnishing an accurate description of the effect of the solvent on the mode. The tNMA dimer frequencies found at 1655 and 1686 cm^−1^ can also be nicely compared with the experimental 1655 cm^−1^ value. A more quantitative agreement with literature data is also achieved in the AcAlaOMe when the cluster representation is used (1658 vs. 1672 cm^−1^) and partially through the dynamic analysis (1680–1723 vs. 1672 cm^−1^). Finally, in the literature it is reported that the tNMA monomer [83] satisfactorily reproduces the experimental 1620 cm^−1^ value. Regarding the amide II, the implicit solvent can more quantitatively catch the solvent blue-shift effect. Indeed, for the dimer case, the amide II is detected at 1529/1556 (A/D) cm^−1^ vs. 1555 cm^−1^ (experimental value). Interestingly, in this case the dynamic picture provides a more quantitative agreement with the experimental frequency (1551 and 1549 cm^−1^ for the acceptor and donor, respectively), in comparison with the monomer case reported in literature [83]. In agreement with the hypothesis that the effect of a poor description of the oxygen lone pairs by the MM picture influences the accurate simulation of the effect of the solvent on the N–H and C–N bonds, in the dimer case the latter is mitigated by the intermolecular interaction between the two monomers. Indeed, since the first perturbation to the amide II is due to the interaction between the two tNMA monomers that is described at the QM level, it could be plausible that the description at a lower level of the solvent does not importantly influence the proper description of the mode. Similarly, also in the AcAlaOMe case, the AII is nicely described by the implicit solvent static, and even better by the dynamic, representations (1493, 1510 vs. 1514 cm^−1^). In both dimer and AcAlaOMe, the static cluster simulations overestimate the blueshifts probably because the interactions with the solvent are too emphasized. Although an experimental value is not available for the amide III mode, it is reasonable that a scenario similar to the amide II would be found. Also, in this case, the cluster picture provides blue-shifted frequencies compared to the C-PCM ones for the dimer case. Regarding the tNMA monomer reported in the literature [83], the cluster representation remains the more accurate (1284, 1306, 1267 for the C-PCM, cluster, and dynamic representations, respectively) vs. 1300 cm^−1^. Again, the effect of the MM representation of water molecules has a more pronounced effect on the correct reproduction of the amide II blue shift. In C-PCM representation of the amide A of the dimer, the frequency associated with the donor is importantly underestimated (3299 vs. 3340 cm^−1^), while the acceptor is too overestimated (3480 vs. 3340 cm^−1^). A more correct representation is provided by the cluster case (3309/3342 vs. 3340 cm^−1^ for the A/D). Of course the experimental data is an average between the two contributions, slightly emphasized in the cluster picture. However, the difficulty in simulating quantitatively the red-shift is possibly due to the intrinsic challenge of simulating the solvent effect by an MM description. Interestingly, for AcAlaOMe, the C-PCM and dynamic descriptions are closer to each other and to reference literature data (3460, 3447 vs. 3464 cm^−1^). Possibly, in this case, smoother specific interactions with the solvent are established. Conversely, the explicit solvent description in the static cluster case provides better frequency values for the tNMA monomer (3340 vs. 3300 cm^−1^). Overall, this analysis demonstrates that a vibrational analysis from AIMD can correctly describe the amide modes vibrational features and their perturbation due to the solvent. In general, explicit solvent modeling can improve the description and interpretation of the modes; however, in some cases, it could cause an overestimation of the solvent effect.

## 3. Materials and Methods

For large polyatomic molecules, i.e. biological macromolecules or condensed phase systems a synergistic vibrational analysis protocol is mandatory, since it becomes not negligible the interplay of strong anharmonicities, thermal effects, mode couplings, and solute–solvent interactions. To address these limitations, we adopt a dual strategy (i) a Hessian-based normal mode analysis, including anharmonic corrections via perturbation theory, and (ii) a time-dependent vibrational analysis, by extracting generalized normal modes (GNMs) by performing ab initio molecular dynamics (AIMD) simulations, capable of capturing anharmonicity and dynamical effects explicitly [83]. To provide additional insights to the dynamical microsolvation, GNMs were also studied by exploiting wavelet-based time resolved vibrational analysis, along with relying on an accurate energy potentials obtained by hybrid implicit/explicit solvation methods enforcing non-periodic boundary conditions (NPBC) [91,110]. Although designed for general treatment of solute–solvent systems to capture transient solute–solvent coupled vibrations, the present approach is here specifically devoted to the study of peptides in water solution. To better highlight the solute–solvent interaction on a molecular size scale, both methods are applied to our model peptide systems in vacuum and aqueous solution.

### 3.1. Hessian-Based Vibrational Analysis

In the harmonic approximation, vibrational frequencies are obtained from the diagonalization of the mass-weighted Hessian matrix, ref. [59] whose elements are the second derivatives of the potential energy with respect to Cartesian atomic displacements. The eigenvalues of the Hessian correspond to the squared harmonic frequencies $\omega_{i}^{2}$, and the eigenvectors define the normal modes (NMs). To improve upon the harmonic approximation, we introduce anharmonic corrections using second-order vibrational perturbation theory (VPT2) [60,61,62,111]. This approach required representative minimum energy structures (it is also commonly called ‘static approach’) and becomes computationally intractable for systems with many degrees of freedom. For these reasons, we limited such analysis on few molecular clusters, whose microsolvation was based on the knowledge acquired from the MD simulations.

### 3.2. Generalized Normal Modes and Time-Resolved Vibrational Analysis

To overcome the limitations of static approaches, we employ a time-dependent method based on AIMD simulations, capable of naturally incorporating finite temperature, anharmonicity, and environmental effects. The method is based on the extraction of generalized normal modes from AIMD trajectories [68,78,79,80,81,82,83,84].

The key assumption here is that GNMs correspond to statistically uncorrelated atomic momenta, and they are obtained diagonalizing the covariance matrix of the mass weighted atomic velocities **K** with elements:

(1) $$K_{ij}=\frac{1}{2}\sqrt{m_{i}m_{j}}\langle \left({\dot{q}}_{i}-\langle {\dot{q}}_{i}\rangle \right)\left({\dot{q}}_{j}-\langle {\dot{q}}_{j}\rangle \right)\rangle$$

where ${\dot{q}}_{i}$ and ${\dot{q}}_{j}$ are the 3N atomic cartesian velocities collected along the trajectory (the explicit dependence upon *t* is not shown for simplicity), $m_{i}$ is the mass associated with Cartesian velocity ${\dot{q}}_{i}$ and 〈〉 denotes an ensemble average, constructed in this work by averaging over time the required quantities over a single collected trajectory. Generalized, normal-like modes are obtained as eigenvectors of the K matrix while the eigenvalues of K correspond to the averaged kinetic energy for each mode.

Generalized mode velocities are calculated at each time step by projecting atomic velocities along each normal-like mode:

(2) $$\dot{Q}\left(t\right)=L^{†}\dot{q}\left(t\right)$$

where L is the unitary transformation matrix which diagonalizes K. Thus, the calculation of generalized modes from the unitary transformation of velocity matrix K (Equation (2)) and the computation of frequencies by the power spectra of velocities projected along the generalized modes, appear to be the most general choice to perform vibrational analysis of fundamental anharmonic vibrations from ab initio trajectories at finite temperature. Therefore, relying on previous studies we are confident that a combination of the velocity-covariance based generalized vibrational modes analysis from AIMD with wavelet theory can be an useful instrument to catch anharmonically coupled solute–solvent vibrational dynamics in condensed phase systems.

Once obtained the GNMs, their power spectrum is generally obtained via the Fourier transform (FT) of their velocity autocorrelation function, *P*^*α*^(*ω*), comparable to infrared, IR, and Raman profiles regardless of their specific selection rules:

(3) $$P^{\alpha }\left(\omega \right)=\int {\langle {\dot{Q}}^{\alpha }\left(\tau \right){\dot{Q}}^{\alpha }\left(t+\tau \right)\rangle }_{\tau }e^{-i\omega t}dt$$

where the superscript α runs over the 3 N generalized normal coordinates, of which the first six account for translational and rotational collective motions.

This method is appropriate for stationary signals, providing an averaged frequency domain representation. However, this representation fails in retaining the temporal evolution of the signals that is mandatory to catch the time-evolution of the vibrational bands. This information can be critical also in stationary cases in terms of both frequency shifts and possible time-dependent couplings with other modes. To resolve the limitations of FT for non-stationary signals, we introduce the continuous wavelet transform (CWT) [72], which enables simultaneous localization in time and frequency. The CWT of a signal C(t) with respect to a wavelet function $\psi_{a,b}\left(t\right)$ is defined as

(4) $$W\left(a,b\right)=\int C\left(t\right)\psi_{a,b}\left(t\right)\;dt$$

with:

(5) $$\psi_{a,b}\left(t\right)={\left|a\right|}^{-1/2}\psi \left(\frac{t-b}{a}\right)$$

Here, *a* is the scale (inversely proportional to frequency) and *b* is the time translation parameter. We use the Morlet wavelet, a complex function composed of a Gaussian-modulated plane wave:

(6) $$\psi \left(t\right)=\pi^{-1/4}e^{i\omega_{0}t}e^{-t^{2}/2}$$

with $\omega_{0}\geq 6$ to satisfy the admissibility condition. This wavelet provides good resolution for oscillatory signals and is well-suited for the analysis of vibrational modes dynamics. To accelerate the computation of the spectra, the CWT is evaluated in the frequency domain using the convolution theorem:

(7) $$W\left(a,b\right)=\sum_{k=0}^{N-1}{\hat{x}}_{k}{\hat{\psi }}^{*}\left(a\omega_{k}\right)e^{i\omega_{k}b\delta t}$$

where *N* is the number of points in the time series, k=0⋯N−1 is the frequency index, ${\hat{x}}_{k}$ is the discrete Fourier transform of the general time series, $\hat{\psi }$ is the Fourier transform of the wavelet function, ${(}^{*})$ indicates the complex conjugate, δt is the sampling time, and $\omega_{k}$ is the angular frequency. The resolution of a wavelet function is determined by the balance between the width in the real space and the width in Fourier space. The shape of the wavelet function should reflect the type of features present in the time series, i.e., a boxcar-like function for sharp peaks or steps in the time series, a damped cosine for smoothly one. The choice of scales (*a* parameters) must be carried out in order to obtain a complete picture of the frequencies. It is computationally affordable to express scale parameter as fractional powers of two:

(8) $$a_{j}=a_{0}2^{j\delta j},j=0,1,...,J$$

(9) $$J=\delta j^{-1}log_{2}\left(N\delta t/a_{0}\right)$$

where $a_{0}$ is the smallest resolvable scale (tipically 2δt), *J* is the largest scale, δj is the scale spacing and δt is the sampling time. For the Morlet wavelet, a δj value of 0.5 still gives adequate sampling in scale; smaller values of δj give finer resolution. The relationship between equivalent Fourier period and the wavelet scale can be derived, for a particularly wavelet function, by substituting a cosine wave into the expression of CWT and computing the scale *a* at which the wavelet power spectrum reaches its maximum. For the Morlet function with $\omega_{0}=6$, this gives a value of λ=1.03*a* where λ is the Fourier period, indicating that the scale is almost equal to the Fourier frequency.

This approach allows for efficient multiresolution analysis of vibrational signals, revealing time-dependent couplings and mode activations that are otherwise inaccessible through standard FT methods. The frequency resolution of the transient vibrational spectra computed via WT for the ground state analysis is approximately 5 cm^−1^ in the AIII region, 8 cm^−1^ for the Amide I, and 15 cm^−1^ in the Amide A range. In summary, by combining conventional Hessian-based methods with AIMD simulations and advanced signal processing techniques, we can achieve a comprehensive view of vibrational properties of the investigated molecules in gas and condensed phase. This hybrid strategy not only captures the fundamental vibrational features but also reveals dynamical effects, mode couplings, and solute–solvent interactions critical to understand molecular behaviors in realistic environments.

### 3.3. Computational Details

#### 3.3.1. Hessian-Based Vibrational Analysis

For the three case studies (tNMA, tNMA dimer through space. and AcAlaOMe), geometry optimization calculations without any constraints, anharmonic frequencies, and IR intensities were performed on isolated systems as well as by using the conductor-like polarizable continuum model (C-PCM) implicit solvent picture at B3LYP/6-31G(d,p) [112,113] theory level using the Gaussian09 quantum chemical package [114]. This level of theory has been extensively validated in the literature for peptide vibrational spectroscopy, and it has been shown to reliably reproduce both qualitative frequency trends and normal mode assignments in small peptides and related model systems [115,116,117,118,119]. The effects of hydrogen bonds on amide modes in the last two systems have been evaluated through full *QM* optimization and anharmonic frequencies calculations on cluster models, including bulk solvent effects via the C-PCM implicit solvent model at the same theory level. In these clusters, water molecules engage hydrogen bonds with both the C=O and the N–H sites. Regarding the number of water molecules included in the clusters, we used the information on the microsolvation provided by the radial distribution functions (RDFs) computed on the AIMD trajectories. In detail, for the dimer case, two water molecules surround the amide C=O moiety of the donor i.e., the tNMA acting as hydrogen bond donor to the other tNMA in the dimer (also referred to as D), and one water molecule surrounds the amide N–H group of the acceptor i.e., the tNMA acting as hydrogen bond acceptor by the other monomer in the dimer (also referred to as A). Moreover, a fourth water molecule surrounds the dimer interface. Regarding AcAlaOMe, two water molecules surround the amide C=O moiety, and one water molecule surrounds the amide N–H group.

#### 3.3.2. Ab-Initio Molecular Dynamics Simulations

The molecular dynamics simulations for the three molecules in the gas phase ran over potentials calculated at the B3LYP/6-31G(d,p) level of theory at a temperature T = 300 K and 0.2 fs as a time step, starting from the minimum energy structure coordinates obtained at the same theory level. The frequency of velocity randomization is variable and depends on the system itself. Initial random velocities in agreement with a temperature of 300 K are employed. The electronic density matrix was computed on-the-fly during the simulation, following the ADMP formalism [65,66,67,120]. The core and valence orbitals were differently weighted during the dynamics with $\mu_{valence}=0.1$ amu for the valence electrons and $\mu_{core}$ obtained according to the tensorial fictitious mass scheme (see Ref. [66]). For isolated tNMA, tNMA dimer, and AcAlaOMe systems, we collected trajectories of 15, 25, and 15 ps, respectively. To disentangle the complex vibrational behavior of the dimer, we performed vibrational analysis on individual monomers extracted sequentially from the trajectory. A custom program developed by some of the authors has been used to carry out the wavelet transform (WT), following the methodology proposed by Torrence and Compo [72]. The WT, performed only for characteristic modes as Amide I, Amide II, Amide III, and Amide A, is reported.

We collected a 16 ps long *QM*/*MM* trajectory with 0.2 fs as time step for the tNMA dimer and AcAlaOMe. The first system was solvated by 361 water molecules, while the second one by 218. Both systems have been enclosed into a spherical cavity with a radius of 14.0 Å and 12.0 Å respectively, enforcing NPBC. The size of the spherical-shaped boxes was adapted to reproduce the RT density of water by taking into account the volume occupied by the solute. All solute molecules have been treated at the full *QM* level, while the solvent were described using a flexible and re-parametrized TIP3P water model [78]. This modified model was specifically designed to better capture vibrational couplings involving both the water bending and the vibrational modes of the solute. The re-parametrization procedure yielded an equilibrium H–O–H angle of 104.52° and a bending force constant of 50 kJ mol^−1^ rad^−2^. The ADMP formalism, previously introduced, is applied. The AIMD simulations, performed under non-periodic boundary conditions, were run using a development version of Gaussian [121]. Concerning the tNMA dimer, we have split and analyzed only the part of the trajectory in which the hydrogen bond between the two monomers survives (11 ps) to try to maximize the capability to catch of the solvent effect on the modes. To quantitatively include the solvent effects, the vibrational analysis of the modes was performed by extracting the velocities of the solute and the closest surrounding water molecules. In particular, for the dimer four and for the AcAlaOMe three, according to the microsolvation analysis performed on the trajectories. Finally, we calculated the distributions of structural parameters for the tNMA dimer and AcAlaOMe derived from AIMD simulations in both the gas and aqueous phases. The distributions were computed with a bin size of 0.001 Å for bond distances and 0.05° for angles and dihedral angles.

## 4. Conclusions

In the present work, we employed a theoretical and computational protocol that combines ab initio molecular dynamics simulations with a multiresolution wavelet-based analysis to perform time-resolved vibrational characterization of peptide model systems in both the gas phase and explicit aqueous solution. Unlike conventional Fourier analysis, the wavelet transform enables simultaneous time–frequency resolution. We demonstrated that the wavelet-based analysis is critical to quantitatively investigate the interplay between molecular structure, solvent environment, vibrational couplings, and frequency shifts also when dealing with stationary signals. The peptide models chosen as case studies to atomistically investigate the solvent effect on main amide modes (amide I, II, III, and A) frequency and composition were tNMA, tNMA dimer, and AcAlaOMe. These systems are representative of typical protein intra- and intermolecular interactions, including those involving water molecules (the cybotactic region). The comparison with the static Hessian-based approach unveiled that the latter is not able to catch the couplings among vibrational bands. Moreover, thanks to the wavelet transform, these coupling and frequency shifts can be located on-the-fly during the time. A dynamic picture of how solvent fluctuations locally affect the structure and the vibrational behavior over time is achieved, demonstrating the importance of a time-dependent description also for stationary signals. The achieved insights highlight that this method represents a powerful and versatile tool for the theoretical exploration of modern vibrational spectroscopy techniques, allowing for the direct connection between vibrational modes and local structural changes.

## Figures and Tables

**Table 1:** Average bond lengths (Å) extracted from AIMD trajectories in gas-phase and aqueous solution. For the tNMA dimer, A and D denote the hydrogen bond acceptor and donor monomers, respectively. Standard deviation is reported in parenthesis. The tNMA parameters in aqueous solution are taken from Ref. [83].

	Gas Phase (AIMD)			Aqueous Solution (AIMD)		
	C=O	C–N	N–H	C=O	C–N	N–H
tNMA	1.224	1.379	1.013	1.245	1.348	1.016
	(0.011)	(0.029)	(0.025)	(0.019)	(0.024)	(0.007)
tNMA dimer	(A) 1.231	(A) 1.368	(A) 1.011	(A) 1.244	(A) 1.351	(A) 1.017
	(0.018)	(0.027)	(0.016)	(0.015)	(0.019)	(0.028)
	(D) 1.228	(D) 1.369	(D) 1.017	(D) 1.243	(D) 1.353	(D) 1.020
	(0.013)	(0.021)	(0.029)	(0.022)	(0.022)	(0.020)
AcAlaOMe	1.227	1.374	1.014	1.239	1.361	1.018
	(0.018)	(0.025)	(0.017)	(0.022)	(0.028)	[0.010)

**Table 2:** Vibrational analysis of the peptide model systems in the gas phase. Frequencies are reported in cm^−1^ and are computed both by Hessian-based VPT2 (static) and AIMD GNM (dynamics) approaches. The results are compared with literature values, including both theoretical predictions (calc) and experimental measurements (exp).

	*Gas Phase*		
	tNMA	tNMA Dimer	AcAlaOMe
**Amide I**			
Static	1757	1743 (A); 1744 (D)	1737
Dynamics	1723	1698 (A); 1715 (D)	1708
Refs	1735 ^*a*^ (calc), 1731 ^*b*^ (exp)	1700 ^*c*^ (exp)	1678, 1665 ^*d*^ (exp)
**Amide II**			
Static	1517	1534 (A); 1537 (D)	1496
Dynamics	1497	1515 (A); 1505 (D)	1470
Refs	1522 ^*a*^ (calc), 1499 ^*b*^ (exp)	1530 ^*c*^ (exp), 1555 ^*e*^ (exp)	/
**Amide III**			
Static	1254	1262 (A); 1259 (D)	1232
Dynamics	1244	1247 (A); 1243 (D)	1215
Refs	1231 ^*a*^ (calc), 1265 ^*b*^ (exp)	1300 ^*c*^ (exp), 1275 ^*c*^ (exp)	/
**Amide A**			
Static	3492	3484 (A); 3383 (D)	3451
Dynamics	3511	3533(A); 3167 (D)	3508
Refs	/	3374 ^*c*^ (exp), 3318 ^*c*^ (exp), 3498 ^*f*^ (exp)	3452 ^*d*^ (exp)

^*a*^ Ref. [108]; ^*b*^ Ref. [87]; ^*c*^ Ref. [100]; ^*d*^ Ref. [23]; ^*e*^ Ref. [101]; ^*f*^ Ref. [103].

**Table 3:** Vibrational analysis of the peptide model systems in aqueous solution. Frequencies are reported in cm^−1^ and are computed both by Hessian-based VPT2 (static) and AIMD GNM (dynamics) approaches. Hessian-based VPT2 is computed employing both full implicit (C-PCM) and hybrid explicit/implicit (clusters) solvation approaches. The results are compared with literature values, including both theoretical predictions (calc) and experimental measurements (exp).

	*Aqueous Solution*		
	tNMA	tNMA Dimer	AcAlaOMe
**Amide I**			
Static (C-PCM)	1728	1683 (A); 1702 (D)	1701
Static (Cluster)	1588	1651 (A); 1617 (D)	1658
Dynamics	1620 ^*e*^	1655 (A); 1686 (D)	1680-1723
Refs	1620 ^*c*^ (exp), 1690 ^*a*^ (calc)	1655 ^*d*^ (exp)	1672 ^*b*^ (calc)
**Amide II**			
Static (C-PCM)	1548	1529 (A); 1556 (D)	1493
Static (Cluster)	1568	1596 (A); 1601 (D)	1629
Dynamics	1510 ^*e*^	1551 (A); 1549 (D)	1510
Refs	1560 ^*c*^ (exp), 1523 ^*a*^ (calc)	1555 ^*d*^ (exp)	1514 ^*b*^ (calc)
**Amide III**			
Static (C-PCM)	1284	1259 (A); 1285 (D)	1231
Static (Cluster)	1306	1312 (A); 1289 (D)	1337
Dynamics	1267 ^*e*^	1294 (A); 1281 (D)	1290
Refs	1300 ^*c*^ (exp), 1272 ^*a*^ (calc)	/	/
**Amide A**			
Static (C-PCM)	3491	3480 (A); 3299 (D)	3460
Static (Cluster)	3340	3309 (A); 3342 (D)	3143
Dynamics	3455	3490 (A); 3423, 3441 (D)	3447
Refs	3300 ^*c*^ (exp)	3340 ^*d*^ (exp)	3464 ^*b*^ (calc)

^*a*^ Ref. [108], ^*b*^ Ref. [100], ^*c*^ Ref. [109], ^*d*^ Ref. [101], ^*e*^ Ref. [83].

## Data Availability

Data are contained within the article and supplementary materials.

## Supplementary Materials

The following supporting information can be downloaded at: https://www.mdpi.com/article/10.3390/molecules30132854/s1. Gas-phase optimized structures of the peptide models. Normalized distributions of selected structural parameters computed for the tNMA dimer and AcAlaOMe systems in the gas phase and aqueous solution. Bond distances of the peptide models under different chemical environment conditions. Schematic representation of the investigated amide vibrational modes. Wavelet spectra computed from AIMD simulations in the gas phase for the tNMA system. Vibrational spectra computed from AIMD simulations for tNMA, tNMA dimer and AcAlaOMe systems. Ref. [122] is cited in Supplementary Materials.

## Abbreviations

The following abbreviations are used in this manuscript:

A	Acceptor
AcAlaOMe	Methyl-2-Acetamidopropanoate
ADMP	Atom-centered density matrix propagation
C-PCM	Conductor-like polarizable continuum model
D	Donor
DFT	Density functional theory
FSRS	Femtosecond Stimulated Raman Spectroscopy
FT	Fourier transform
GNM	Generalized normal modes
IR	Infrared
MD	Molecular dynamics
MM	Molecular mechanics
NM	Normal modes
NPBC	Nonperiodic boundary conditions
PT2	Perturbation theory to second order
QM	Quantum mechanics
RDF	Radial distribution function
tNMA	trans-N-Methyl-Acetamide
VPT2	Second-order vibrational perturbation theory
WT	Wavelet transform
