# Supplementary material for: Unveiling Vibrational Couplings in Model Peptides in Solution by a Theoretical Approach
Source: Molecules. 2025 Jul 4;30(13):2854. doi: 10.3390/molecules30132854 (PMC12251535; doi:10.3390/molecules30132854)
Supplement: Supplementary file 1 [file molecules-30-02854-s001.zip › molecules-3675290-supplementary.pdf]

Supplementary Material: Unveiling Vibrational Couplings in Model Peptides in Solution by a Theoretical Approach

Federico Coppola,[1] Fulvio Perrella,[1] Alessio Petrone,[2,1,3] Greta Donati,[4,a)] Luciana Marinelli,[4] and Nadia Rega[2,1,3,b)]

[1)] *Scuola Superiore Meridionale, Largo San Marcellino 10, I-80138 Napoli, Italy.*

[2)] *Department of Chemical Sciences, University of Napoli Federico II, Complesso Universitario di M.S.Angelo, I-80126 Napoli, Italy.*

[3)] *Istituto Nazionale Di Fisica Nucleare, sezione di Napoli, Complesso Universitario di M.S.Angelo ed. 6, I-80126 Napoli, Italy.*

[4)] *Department of Pharmacy, University of Napoli Federico II, Via Domenico Montesano 49, 80131 Napoli, Italy.*

(Dated: June 8, 2025)

[a)]Electronic mail: greta.donati@unina.it

[b)]Electronic mail: nadia.rega@unina.it

S1. CONTENTS

Figure S1: Gas-phase optimized structures of the peptide models: tNMA, tNMA dimer, and AcAlaOMe.

Figure S2: Normalized distributions of selected structural parameters computed from the 25 ps AIMD trajectory of the tNMA dimer in gas-phase.

Figure S3: Normalized distributions of selected structural parameters computed from the 15 ps AIMD trajectory of the tNMA dimer in aqueous solution.

Figure S4: Normalized distributions of selected structural parameters computed from the 15 ps AIMD trajectory of the AcAlaOMe system in gas-phase.

Figure S5: Normalized distributions of selected structural parameters computed from the 15 ps AIMD trajectory of the AcAlaOMe system in aqueous solution.

Table S1: Bond distances of the peptide models tNMA, tNMA dimer (D, donor and A, acceptor), and AcAlaOMe under different chemical environment conditions.

Figure S6: Schematic representation of the investigated amide vibrational modes.

Figure S7: Vibrational spectra obtained from the Fourier transform of velocity–velocity autocorrelation functions computed from AIMD simulations in the gas phase for the tNMA system.

Figure S8: Time–frequency vibrational spectra obtained from the Wavelet transform of velocity–velocity autocorrelation functions computed from AIMD simulations in the gas phase for the tNMA system.

Figure S9: Vibrational spectra obtained from the Fourier transform of velocity–velocity autocorrelation functions computed from AIMD simulations in the gas phase for the $tNMA_D$ hydrogen-bond donor monomer.

Figure S10: Vibrational spectra obtained from the Fourier transform of velocity–velocity autocorrelation functions computed from AIMD simulations aqueous solution for the $tNMA_D$ hydrogen-bond donor monomer.

Figure S11: Vibrational spectra obtained from the Fourier transform of velocity–velocity autocorrelation functions computed from AIMD simulations in the gas phase for the $tNMA_A$ hydrogen-bond acceptor monomer.

Figure S12: Vibrational spectra obtained from the Fourier transform of velocity–velocity

autocorrelation functions computed from AIMD simulations aqueous solution for the $tNMA_A$ hydrogen-bond donor monomer.

Figure S13: Vibrational spectra obtained from the Fourier transform of velocity–velocity autocorrelation functions computed from AIMD simulations in the gas phase for AcAlaOMe system.

Figure S14: Vibrational spectra obtained from the Fourier transform of velocity–velocity autocorrelation functions computed from AIMD simulations in the aqueous solution for AcAlaOMe system.

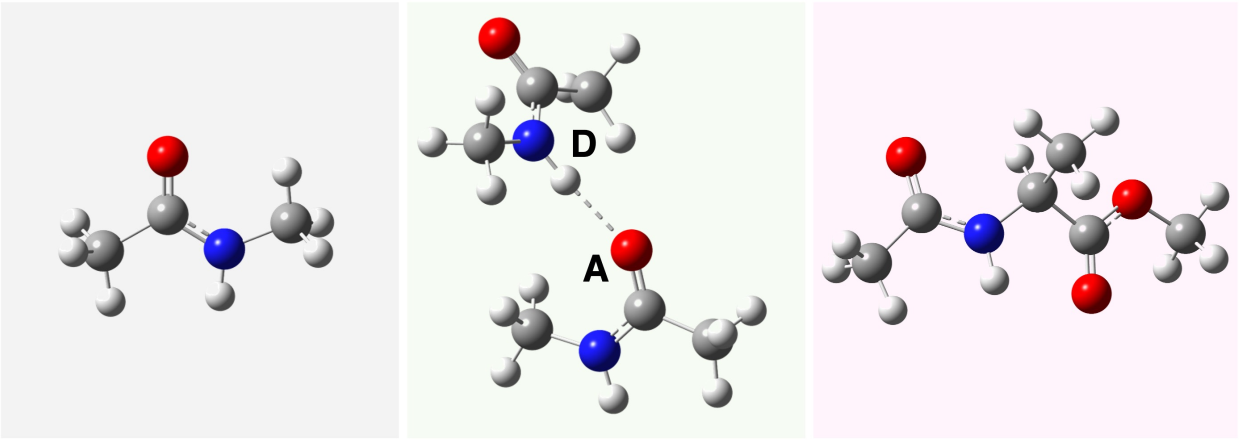

Figure S1. Gas-phase optimized structures of the peptide models at the B3LYP/6-31G(d,p) level. From left to right: trans-N-methylacetamide (tNMA), its hydrogen-bonded dimer (D: donor, A: acceptor), and methyl-2-acetamidopropanoate (AcAlaOMe). Color code: carbon (gray), hydrogen (white), nitrogen (blue), oxygen (red).

S2. STRUCTURAL CHARACTERIZATION IN GAS AND CONDENSED PHASES FROM AIMD SIMULATIONS

In Figure S2, we present the distributions of selected structural parameters derived from 25 ps AIMD trajectories of the tNMA dimer sampled in the gas phase. The distribution of structural parameters for the secondary amides of monomer D reveals a sharp C=O bond length centered at 1.228 Å, with the C–N bond ranging from 1.288 to 1.460 Å (mean, 1.369 Å). The The N–H bond exhibits a bimodal distribution, with an average value of 1.017 Å; the absence of a single dominant peak is due to dipolar interactions involving the N–H moiety.[1] For the A subunit, influenced by interaction with monomer D, the C=O distribution slightly broadens compared to the previous case (1.173-1.296 Å), peaking at 1.231 Å. Effects of the hydrogen bond are evident in the C–N distribution, which peaks at 1.368 Å. The N–H bond length distribution also shows a bimodal pattern centered around 1.011 Å. To further characterize the hydrogen-bond interaction, we also examine the $H_D \cdots O_A$ distance and $(H–N)_D$-O_A angle. The hydrogen bond length distribution ranges from 1.600 Å to 3.211 Å, with a peak occurrence around 2.235 Å. Throughout the simulation, the two monomers undergo relative positional changes; notably, the $(H–N)_D$-O_A angle angle spans a wide range of values, with a maximum occurrence near 16.91°, indicating persistence of the hydrogen bond during AIMD simulations. To assess the conformational space accessible to the tNMA dimer, we calculate the $C–N_D \cdots C–N_A$ improper dihedral angle to evaluate the orientation between the two molecular planes. In the gas-phase simulation, the two monomers can easily orient themselves with respect to each other, as evidenced by a distribution that spans the entire range of ±180°, with a peak centered around 60°.

When the tNMA dimer is placed in water, significant structural changes occur in both subunits (see Figure S3). For the tNMA donor, the distribution of C=O is centered at 1.243 Å and is wider than the gas-phase (0.930 Å vs 0.132 Å); the same holds for the C–N bond (0.171 Å vs 0.204 Å) which shrinks in water (1.353 Å, on average) while the N–H bond exhibits a bimodal distribution and undergoes an elongation of 0.004 Å(1.020 Å). The different broadening observed, arises from intermolecular interactions with surrounding water molecules around the carbonyl group, which affect the electronic structure of the secondary amide, as well as from intramolecular interactions with the acceptor tNMA unit, from the N–H side. In contrast, the acceptor monomer — where the C=O group participates in two

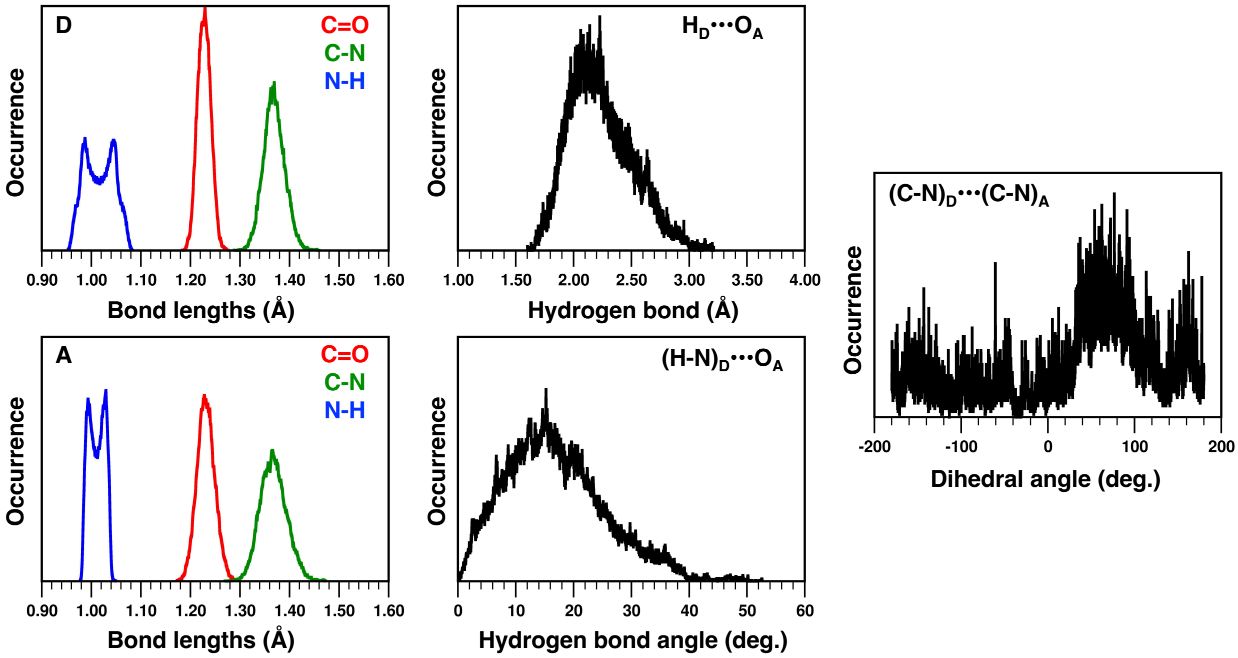

Figure S2. Normalized distributions of selected structural parameters computed from the 25 ps AIMD trajectory of the *t*NMA dimer in gas-phase. The panels show the probability distributions of bond lengths (C=O, C–N, and N–H) for both donor (D) and acceptor (A) subunits, hydrogen bond distances ($H_D \cdots O_A$) and angles $(H\text{-}N)_D \cdots O_A$, and the $C\text{–}N_D \cdots C\text{–}N_A$ improper dihedral angle, used to characterize the mutual orientation of the two molecular planes. Bond lengths are reported in Å, while angles and dihedrals are in degrees. Distributions were computed using a bin size of 0.001 Å for distances and 0.05° for angles and dihedrals.

hydrogen bonds — for both the C=O and C–N bond lengths, the distributions are slightly narrower and centered around average values of 1.244 Å and 1.351 Å, respectively, compared to the gas-phase. This suggests that intermolecular interactions with another peptide-like system have a somewhat reduced structural impact compared to the solvent-induced perturbations experienced by an exposed carbonyl group. The N–H bond in the acceptor unit displays a broader, still bimodal, distribution centered around 1.017 Å, indicating enhanced structural flexibility when the N–H moiety is exposed to the aqueous environment rather than forming a hydrogen bond with a peptide partner. The two descriptors chosen to characterize the intermolecular hydrogen bond between the tNMA subunits further support this picture. Specifically, the hydrogen bond distance ($H_D \cdots O_A$) distribution becomes

narrower in water and is centered at 2.044 Å, while the corresponding $(\text{H–N})_D$-O_A angle shows a sharper, peaked distribution with a mean value of 13.11°. These features indicate a strengthening of the hydrogen bond in solution, with the two subunits adopting a more compact and stable arrangement. Finally, the C–$\text{N}_D\cdots$C–N_A improper dihedral angle, used to evaluate the relative orientation between the two molecular planes, displays a significantly narrower distribution in water compared to the gas phase. Notably, it exhibits two dominant peaks centered at 90° and 120°. This finding reveals a loss of conformational flexibility in the aqueous phase, suggesting that the chemical environment - particularly the cybotactic region - plays a crucial role in guiding and modulating the conformational space accessible to the dimer.

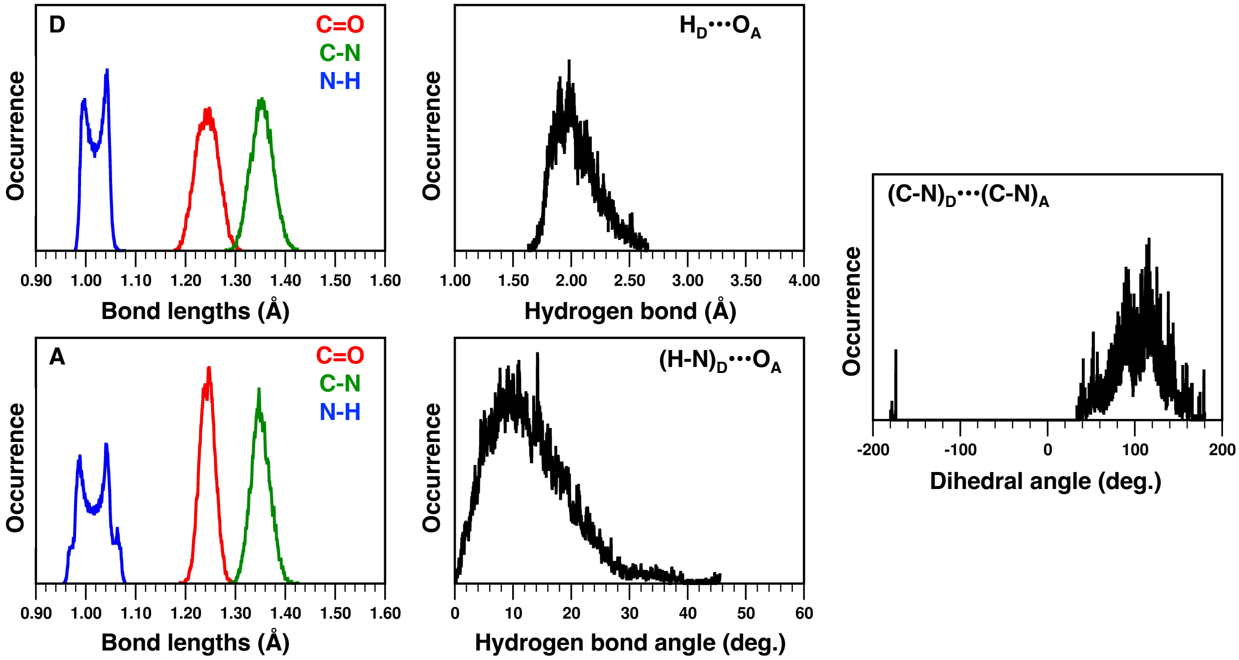

Figure S3. Normalized distributions of selected structural parameters computed from the 15 ps AIMD trajectory of the *t*NMA dimer in aqueous solution. The panels show the probability distributions of bond lengths (C=O, C–N, and N–H) for both donor (D) and acceptor (A) subunits, hydrogen bond distances ($\text{H}_D\cdots\text{O}_A$) and angles $(\text{H-N})_D\cdots\text{O}_A$, and the $\text{CN}_D\cdots\text{CN}_A$ improper dihedral angle, used to characterize the mutual orientation of the two molecular planes. Bond lengths are reported in Å, while angles and dihedrals are in degrees. Distributions were computed using a bin size of 0.001 Å for distances and 0.05° for angles and dihedrals.

In Figure S4, we report the distribution of key structural parameters obtained from AIMD simulations of AcAlaOMe in the gas phase. The C=O bond length exhibits a distribution typical of a free carbonyl group, with an average value of 1.227 Å. The C–N bond length shows a narrow distribution centered at 1.374 Å, while the N–H bond displays a bimodal distribution ranging from 0.983 to 1.049 Å, with an average value of 1.014 Å. To describe the conformational behavior of AcAlaOMe, we considered the improper dihedral angle defined by the N–H–C=O_{ester} fragment. Its distribution spans from −59.48° to −38.11°, suggesting a fluxional behavior in the gas phase, where the N–H and C=O groups oscillate reciprocally. The maximum of the distribution is located around -8.32°, indicating that, on average, the peptide adopts a nearly linear conformation.

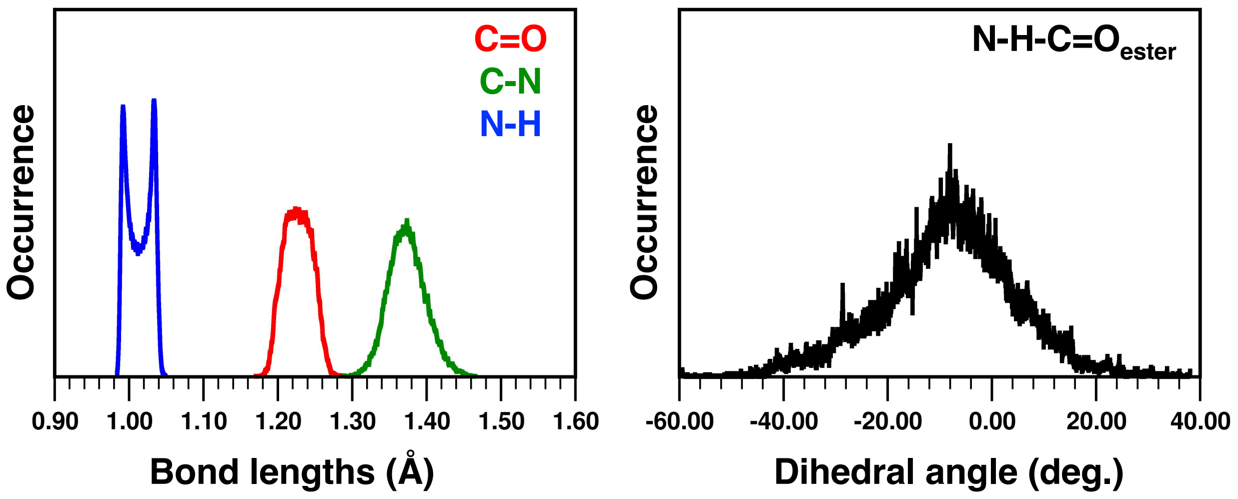

Figure S4. Normalized distributions of selected structural parameters computed from the 15 ps AIMD trajectory of the AcAlaOMe system in gas-phase. The panels show the probability distributions of bond lengths (C=O, C–N, and N–H) and the N-H-C=O_{ester} improper dihedral angle, used to characterize the conformational variability. Bond lengths are reported in Å, while angles and dihedrals are in degrees. Distributions were computed using a bin size of 0.001 Å for distances and 0.05° for angles and dihedrals.

In aqueous solution, the structural parameters of AcAlaOMe show significant deviations from those observed in the gas phase, see Figure S5. The C=O bond length ranges from 1.172 to 1.313 Å, with an average value of 1.239 Å, indicating a broader and slightly shifted distribution compared to the gas-phase value of 1.227 Å. This broadening is consistent with enhanced fluctuations due to solute-solvent interactions involving the carbonyl oxygen.

The C–N bond length also displays a wider distribution in water (1.272–1.471 Å) and a slightly shorter average value of 1.361 Å, compared to 1.374 Å in the gas phase. This behavior reflects an increased contribution of the resonance form with C=N double-bond character, leading to a shorter and more sp^2-like C-–N bond. The N-–H bond exhibits a distribution between 0.987 and 1.053 Å and an average of 1.018 Å, indicating a slight elongation compared to the gas-phase average (1.014 Å), likely due to hydrogen bonding with surrounding water molecules. To evaluate the conformational flexibility of AcAlaOMe in solution, we examined the distribution of the improper dihedral angle N-H-C=O_{ester}, which spans a range of -132.17° to 21.94°). The average value of -53.18° suggests a deviation from the nearly linear conformation observed in vacuo (mean -8.32°), indicating that solvent interactions induce a more flexible and distorted geometry around the α-carbon group.

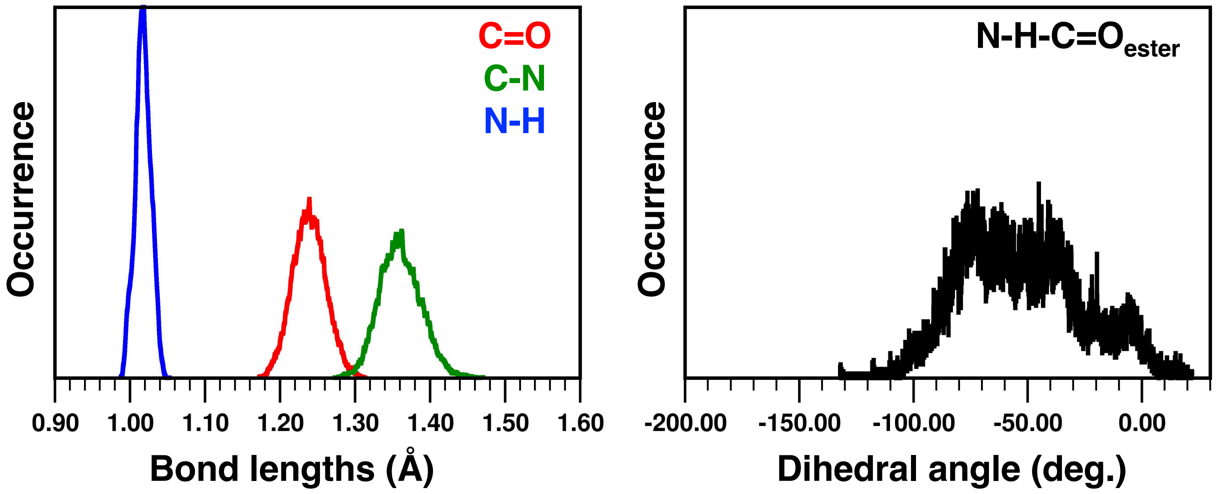

Figure S5. Normalized distributions of selected structural parameters computed from the 15 ps AIMD trajectory of the AcAlaOMe system in aqueous solution. The panels show the probability distributions of bond lengths (C=O, C–N, and N–H) and the N-H-C=O_{ester} improper dihedral angle, used to characterize the conformational variability. Bond lengths are reported in Å, while angles and dihedrals are in degrees. Distributions were computed using a bin size of 0.001 Å for distances and 0.05° for angles and dihedrals.

S3. STRUCTURAL CHARACTERIZATION IN GAS AND CONDENSED PHASES FROM STATIC CALCULATIONS

System	C=O	C–N	N–H	C=O–H_{Wat}	C=O–O_{Wat}	(N)–H–O_{Wat}	N(–H)–O_{Wat}
**tNMA**	1.225<br>*1.235*<br>**1.255**	1.369<br>*1.358*<br>**1.338**	1.008<br>*1.009*<br>**1.024**	**1.830**<br>**1.833**	**2.806**<br>**2.805**	**1.887**	**2.909**
**tNMA$_D$**	1.228<br>*1.238*<br>**1.254**	1.366<br>*1.352*<br>**1.340**	1.016<br>*1.020*<br>**1.022**	**1.836**<br>**1.826**	**2.814**<br>**2.802**	–<br>–<br>–	–<br>–<br>–
**tNMA$_A$**	1.232<br>*1.242*<br>**1.253**	1.359<br>*1.348*<br>**1.336**	1.001<br>*1.009*<br>**1.023**	**1.811**	**2.787**	**1.873**	**2.896**
**AcAlaOMe**	1.227<br>*1.235*<br>**1.250**	1.364<br>*1.357*<br>**1.344**	1.011<br>*1.011*<br>**1.025**	**1.855**<br>**1.841**	**2.825**<br>**2.815**	**1.896**	**2.887**

Table S1. Bond distances (in Å) of the peptide models tNMA, tNMA dimer (D, donor and A, acceptor), and AcAlaOMe under different chemical environment conditions. The gas phase values are reported in regular font, C-PCM implicit solvent in italic, and hybrid implicit/explicit solvation model in bold.

A detailed analysis of the main structural parameters obtained from geometry optimizations under different chemical environments (reported in Tab. S1) provides useful insight into the interplay between the intermolecular interactions, the hydrogen bond with surrounding water molecules, and local geometry in peptide models. For both the tNMA dimer and AcAlaOMe, additional structural features—such as intermolecular angles and specific dihedral variations—are discussed in the following text to further highlight solvent-induced conformational changes. The optimized geometry are sketched in Figure 1. The strength and geometry of the intermolecular hydrogen bond between the donor N–H and the acceptor C=O group in the tNMA dimer is discussed and analyzed through the $H_D \cdots O_A$ distance and the H–$N_D \cdots O_A$ angle considering gas-phase and aqueous solution. For this latter case, an additional geometric parameter that reflects the spatial organization of the tNMA dimer

is the dihedral angle between the molecular planes of the two subunits - approximate here as the $(\mathrm{C–N})_D$–$(\mathrm{C–N})_A$ improper dihedral angle. Similarly, for AcAlaOMe possible conformational changes induced by the chemical environment were assessed by analyzing the N–H–C=O_{ester} dihedral angle.

In the gas phase, all the models exhibit characteristic bond lengths for the peptide backbone: the C=O is consistently found around 1.225-1.232 Å indicating a strong carbonyl character; the C–N bond between 1.359-1.369 Å; and the N–H bonds fall within the expected range for secondary amides (1.001-1.016 Å), with slightly longer value in the donor monomer (tNMA_D) due to the hydrogen bond with the tNMA_A carbonyl. The hydrogen bond in the tNMA dimer is already wellformed, with an $\mathrm{H}_D \cdots \mathrm{O}_A$ distance of 1.979 Å and an almost liner H–$\mathrm{N}_D \cdots \mathrm{O}_A$ angle (5.51°), reflecting a favoreble, strong through space interaction. The interplanar dihedral angle computed for the tNMA dimer is approximately -91°, indicating a near-perpendicular arrangement that minimizes the steric repulsion and allows for an optimal hydrogen bond. Fo AcAlaOMe, the N–H–C=O_{ester} dihedral angle is 3.71°, indicating that the backbone is nearly planar.

Upon introduction of implicit solvation through C-PCM model, the structural parameters undergo a systematic variation. The C=O bonds elongate slightly by ∼0.01 Å, suggesting increased polarization due to the dielectric continuum. Simultaneously, the C–N bond sohrten of the same amount, cosistent with an increase in double bond character due to *resonance* stabilization in the aqueous environment. The N–H distances also increase by 0.01 Å, particularly for the donor subunit (1.020 Å), reflecting a weakening of the bond in the presence of polarizable medium. In this case the hydrogen bond in the tNMA dimer becomes slightly shorter (1.906 Å) and more linear (4.56°), suggesting a stabilization of the D-A interaction due to the presence of polar environment. Additionally, the $(\mathrm{C–N})_D$–$(\mathrm{C–N})_A$ improper dihedral angle increases up to -112.6°, reflecting a pronounced twist in the relative arrangement of the monomers. Compared to the gas-phase, the N–H–C=O_{ester} angle in AcAlaOMe increases to 7.04° in C-PCM, indicating a modest solvent-induced conformational change.

In the hybrid solvation model that combines the explicit water molecules enclosed in the C-PCM cavity, more pronounced structural changes are observed, especially for structural moieties involved in hydrogen bonding. The C=O bond elongate further, reaching value around 1.250 Å, especially when the carbonyl oxygen acts as a hydrogen bond acceptor

from water. The C–N bond further shortens (e.g., 1.336 Å in the $tNMA_A$) increasing the zwitterionic-like resonance structure. The most significant increase is computed for the N–H bond lengths, which reach up to 1.025 Å, particularly when engaged in hydrogen bond either with water or carbonyl oxygen of another peptide unit as in folded structures. For the tNMA dimer surrounded by water molecules, the $H_D \cdots O_A$ distance reduces to 1.894 Å and the H–$N_D \cdots O_A$ angle tightens to 4.46°, indicating an even stronger and better directed hydrogen bond. In the cluster model, hydrogen bonding between the polar groups of the AcAlaOMe backbone and surrounding water molecules promotes a more twisted geometry that enhances solute–solvent stabilization. This results in the most significant conformational change observed, with the N–H–C=O_{ester} dihedral angle increasing up to 25.62°. Additional interatomic distances reported for the cluster models—constructed based on the radial distribution function (RDF) analysis from AIMD nPBC simulations in aqueous solution—offer deeper insight into the hydrogen bonding interactions. The observed C=O$\cdots H_{Wat}$ and N—H$\cdots O_{Wat}$ distances, ranging approximately from 1.8 to 2.9 Å, confirm the establishment of stable hydrogen-bond networks between the peptide and surrounding water molecules.

S4. HESSIAN-BASED ANALYSIS OF PEPTIDE VIBRATIONAL MODES

The vibrational features of peptides and proteins, particularly within the spectral range of 1000-4000 cm^{-1}, are crucial for understand their structural dynamics, being highly sensitive to both microsolvation and conformational changes. In the following section, we present a detailed analysis of these vibrational features in both gas phase and aqueous solution, as obtained from Hessian-based calculations (VPT2) and ab-initio molecular dynamics simulations.

Our discussion focuses on the characteristic secondary amide modes-Amide I (AI), Amide II (AII), Amide III (AIII), and Amide A (AA)-as illustrated in Figure S6.

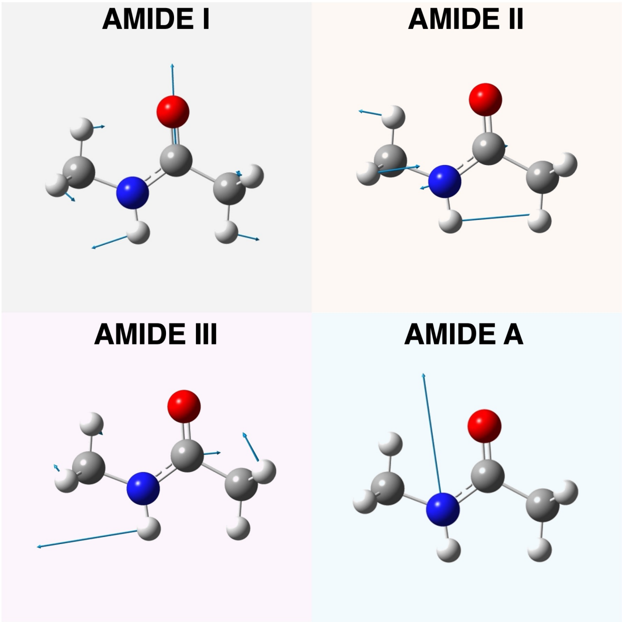

Figure S6. Schematic representation of the investigated amide modes. Amide I (predominantly C=O stretching), Amide II (primarily N–H bending coupled with C–N stretching), Amide III (complex combination of C–N stretching and N–H bending), and Amide A (N–H stretching vibration). The normal mode displacement vectors are shown in blue arrows representation.

Tables 2 and 3 summarized the corresponding vibrational frequencies computed in the gas phase and aqueous solution (using a fully implicit solvent model and a hybdri explict/implicit solvation scheme), respectively, alongside literature comparisons, including both theoretical and experimental values.

In the gas phase, the AI mode (mainly associated to the C=O stretching) exhibits values of 1757 cm^{-1} for tNMA, 1743-1744 cm^{-1} for the tNMA dimer, with negligible splitting between the hydrogen bond donor (D) and acceptor (A) subunits, and 1737 cm^{-1} for AcAlaOMe. Upon solvation, marked frequency red-shift are computed, particularly in the tNMA dimer. Using a fully implicit solvent model, the AI frequency drops to 1728 cm^{-1} for tNMA and to 1702 cm^{-1} (D) and 1683 cm^{-1} (A) for the dimer, highlighting a greater perturbation in the A-moiety where hydrogen bond weakens the C=O bond more effectively. The AI mode of AcAlaOMe red-shifts of 36 cm^{-1} in implicit solvent. When hybrid implicit/explicit solvation is considered, the red-shift becomes even more significant: for tNMA the AI mode red-shifts of 169 cm^{-1}; in the tNMA dimer the AI reaches 1651 cm^{-1} (A) and 1617 cm^{-1} (D). These large frequency shift ($\sim$ 127 cm^{-1} for D subunit) reflects the *cumulative* effects of direct hydrogen bond and solute-solvent interactions on the C=O group. The data further suggest that when C=O is involved in a hydrogen bond with NH group (acts as acceptor), it is less affected by solvent interactions and hence the frequency reds-hift is reduced.

The AII mode, which involves the C–N stretching coupled to the N–H in plane bending, also shows a frequency shift due to the chemical environment changes. In the gas phase, for tNMA and its dimer the AII is peaked at 1517 cm^{-1} and 1534 (A)-1537 (D) cm^{-1}, respectively. When the solvent is accounted in the model as C-PCM, the mode undergoes a slight blue-shift for tNMA (+31 cm^{-1}) but shows a divergent behaviour in the tNMA dimer, with the donor subunit blue-shifting of 19 cm^{-1} compared to the gas phase, while the A-monomer drops to 1529 cm^{-1}. Interestingly, in the hybrid implicit/explicit solvent model, the AII frequency computed for tNMA and both donor/acceptor in the dimer shift further upward of 51, 62 and 64 cm^{-1}, respectively. In contrast, for AcAlaOMe the effects of aqueous environment is actually negligible on AII mode, 1493 cm^{-1} (C-PCM) compared to the gas phase (1496 cm^{-1}). A strong shift towards higher frequency (1629 cm^{-1}) is obtained only when the secondary amide moiety is surrounded by explicit water molecules.

The AIII mode - composed by a more complex combination of C–N stretch and H-N-C bending - exhibits only moderate solvent sensitivity. Compared to the gas-phase value of 1254 cm^{-1} in tNMA, a slight blue-shift of 30 cm^{-1} is observed in implicit solvent, increasing to 52 cm^{-1} in hybrid cluster model. The effects of explicit solvation (or chemical environment changes in general) can be more effectively rationalized by examining the tNMA dimer considering the AII as well as the AIII mode. This model system offer a unique opportunity

to distinguish which part of the secondary amide moiety is more susceptible to hydrogen bonding, either with another peptide chain - mimicking intramolecular interactions in folded peptides - or with surrounding water molecules. By comparing the vibrational shifts of the donor and the acceptor monomers in different solvation environments, it become evident that vibrational modes act differently depending on the nature and also the directionality of the hydrogen bond. Such analysis of AII and AIII modes provides useful insights into the relative sensitivity of backbone vibrations to intra- versus intermoleculear hydrogen-bond interactions, offeering a molecular-level understanding of solvation effects on peptide structure and dynamics. In the gas phase the computed frequencies are nearly identical (1247 cm^{-1} for the acceptor and 1243 cm^{-1} for the donor) indicating a symmetric behavior in absence of solvent. However upon solvation with an implicit model the donor experiences a blue-shift of 42 cm^{-1}, while the acceptor shows a much smaller increase of only 12 cm^{-1}. This difference arises from the localized nature of the N-H$\cdots$O=C hydrogen bond, which primarily stiffs the H-N-C bending component. When explicit water molecules are introduced around the tNMA dimer, a further differentiation in the vibrational landscape of D and A subunits is observed. Notably, the acceptor monomer undergoes a substantial blue-shift of 65 cm^{-1}, while the AIII computed for the donor is 46 cm^{-1}. This inversion of trends suggests that the vibrational behavior of the AIII mode in the acceptor is now more significantly affected by direct interactions with the solvent. In particular the hydrogen bond between the solvent water molecules and the C=O and N–H groups appears to increase the C–N force constant. In contrast, the donor remains primarily influenced by the intermolecular N-H$\cdots$O=C interaction, which is less perturbed by solvent effects. AcAlaOMe follows a similar trend: the AIII mode is unperturbed by implicit solvent (1232 cm^{-1} in gas phase and 1231 cm^{-1} in C-PCM), shifting to higher frequency at 1337 cm^{-1} in the hybrid cluster model.

The AA mode, dominated by the N–H stretching, is the most sensitive to hydrogen bond and shows largest frequency shift upon solvation. In the gas phase, frequencies are in the same spectral range across all systems: 3492 cm^{-1} for tNMA, 3484 cm^{-1} (acceptor) / 3383 cm^{-1} (donor) in the tNMA dimer and 3451 cm^{-1} in AcAlaOMe. The significant red-shift of 100 cm^{-1} observed for the donor in the tNMA dimer, reflects the formation of a stronger intermolecular hydrogen bond and a concomitant weakening of the N–H bond which reduce its vibrational frequency. When the implicit solvation is introduced, this trend is further accentuated. The donor frequency drops to 3299 cm^{-1}, indicating enhanced bond weaken-

ing in the presence of the polarizable continuum, which likely reinforces the intermolecular hydrogen bond (passing from 1.979 Å in gas phase to 1.906 Å in C-PCM for the (N-)H$\cdots$O(=C), as discussed in the previous Section S3) by stabilizing the charge-separated resonance structures. In contrast, the acceptor frequency remains nearly unchanged at 3480 cm^{-1}, suggesting minimal interaction with the solvent field for the non-hydrogen- bonded N–H group. Interestingly, for the hybrid implicit/explicit solvent model, the trend inverts: the AA of the donor rises to 3342 cm^{-1}. The modest blue shift suggests that the original intermolecular hydrogen bond is partially disrupted or weakened by the presence of competing interactions with the explicit water molecules. As a result, the N–H bond becomes somewhat less polarized, leading to a partial restoration of bond strength and a corresponding increase in vibrational frequency. The AA computed for the acceptor undergoes a significant red-shift of 3309 cm^{-1} indicating that the N–H group of the acceptor is now strongly engaged in hydrogen bonding with surrounding water molecules, which weakens the N–H bond and lowers its stretching frequency. For AcAlaOMe, the frequency associated to the AA mode in the gas phase is calculated at 3451 cm^{-1}, upon introducing the solvent as polarizable continuum model, the frequency shift negligibly to 3460 cm^{-1}. However, the inclusion of explicit water molecules in the model leads to a marked redshift dropping to 3143 cm^{-1}. This large shift implies the formation of hydrogen bonds between the N–H group and the surrounding water molecules, which weakens the N–H bond and lowers its vibrational frequency. The significant difference between the gas phase and cluster model results highlights the strong impact of explicit solvation on the vibrational properties of the N–H group, with the solvent inducing a more pronounced perturbation than the implicit C-PCM model.

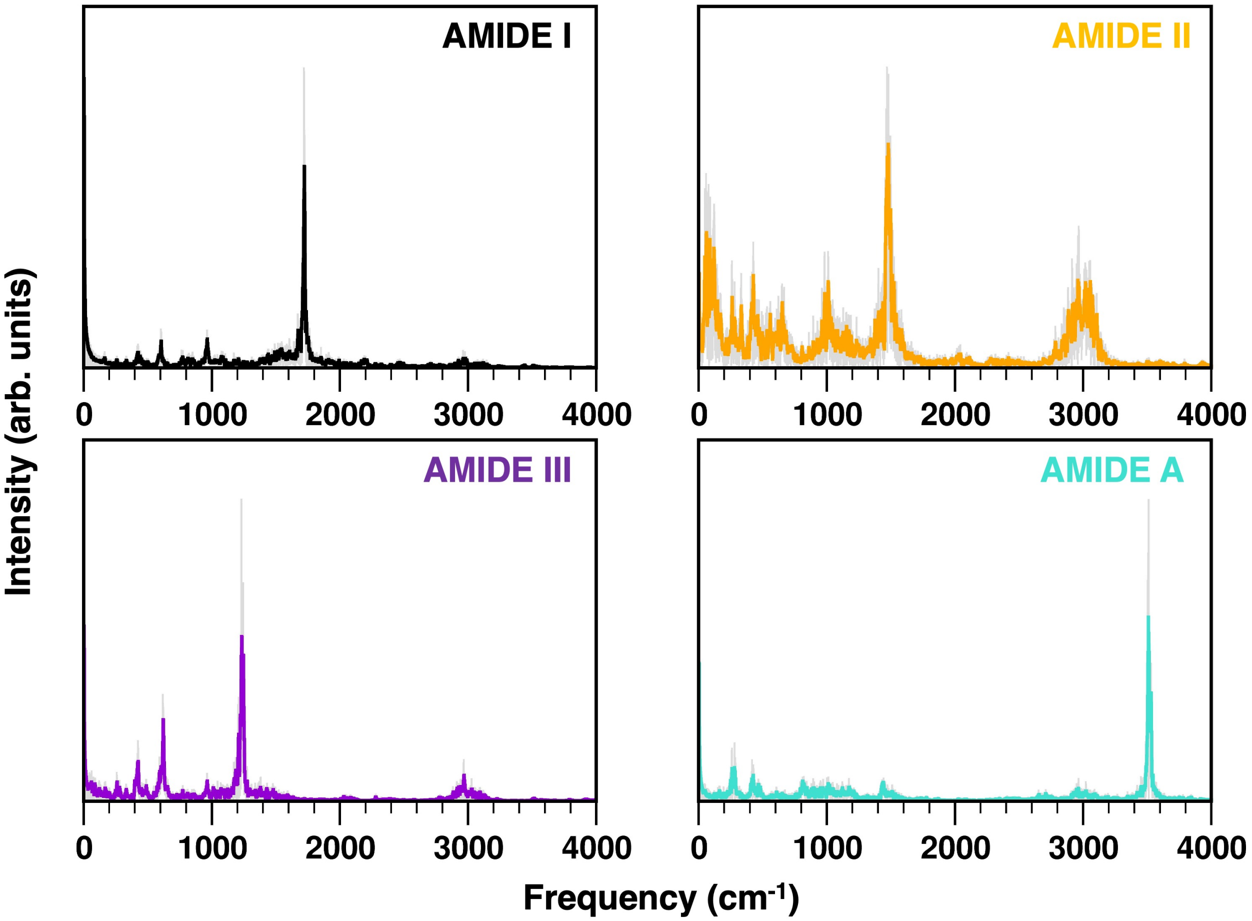

Figure S7. Vibrational spectra obtained from the Fourier transform of velocity–velocity autocorrelation functions computed from AIMD simulations in the gas phase for the tNMA system. Each panel highlights the spectral contribution of a specific amide band: **Amide I** in the top left, **Amide II** in the top right, **Amide III** in the bottom left, and **Amide A** in the bottom right. Intensities are normalized and reported in arbitrary units as a function of frequency (cm^{-1}).

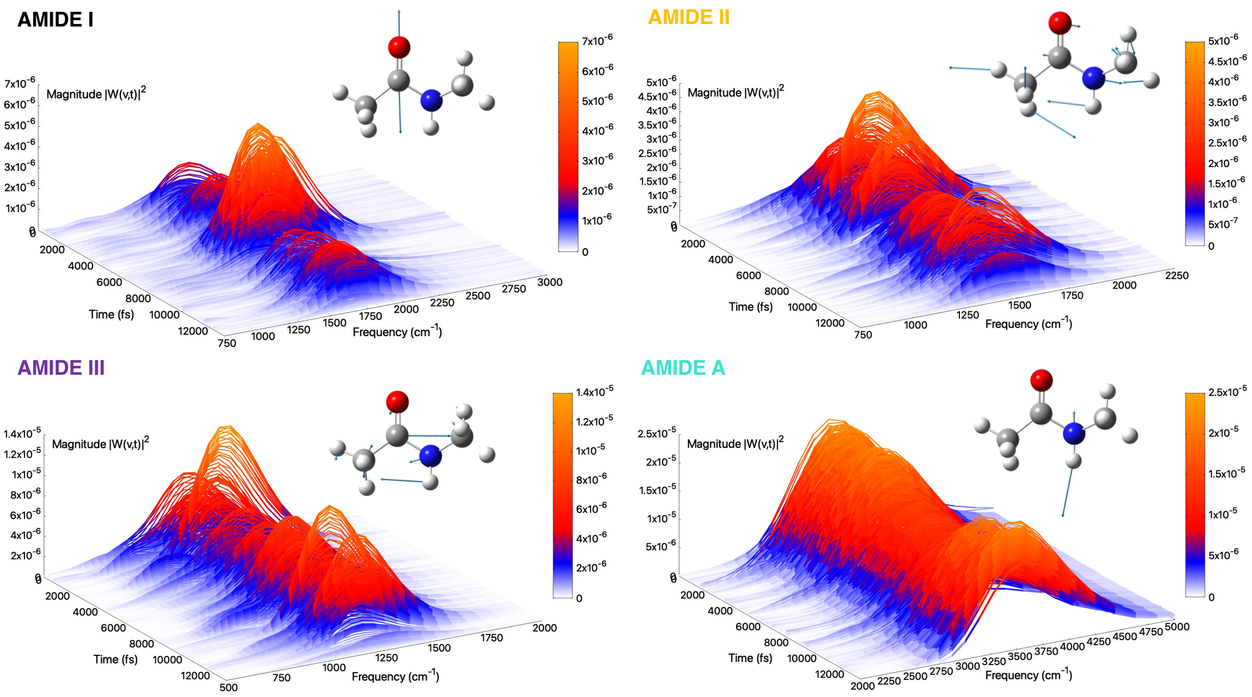

Figure S8. Time–frequency vibrational spectra obtained from the Wavelet transform of velocity–velocity autocorrelation functions computed from AIMD simulations in the gas phase for the tNMA system. Each panel highlights the contribution of a specific amide band: **Amide I** in the top left, **Amide II** in the top right, **Amide III** in the bottom left, and **Amide A** in the bottom right. The corresponding generalized normal mode compositions extracted from the trajectory are also shown to the right of each spectrum. The x-axis represents time, the y-axis frequency (cm^{-1}), and the intensity of the power spectrum is reported in the color scale (z-axis).

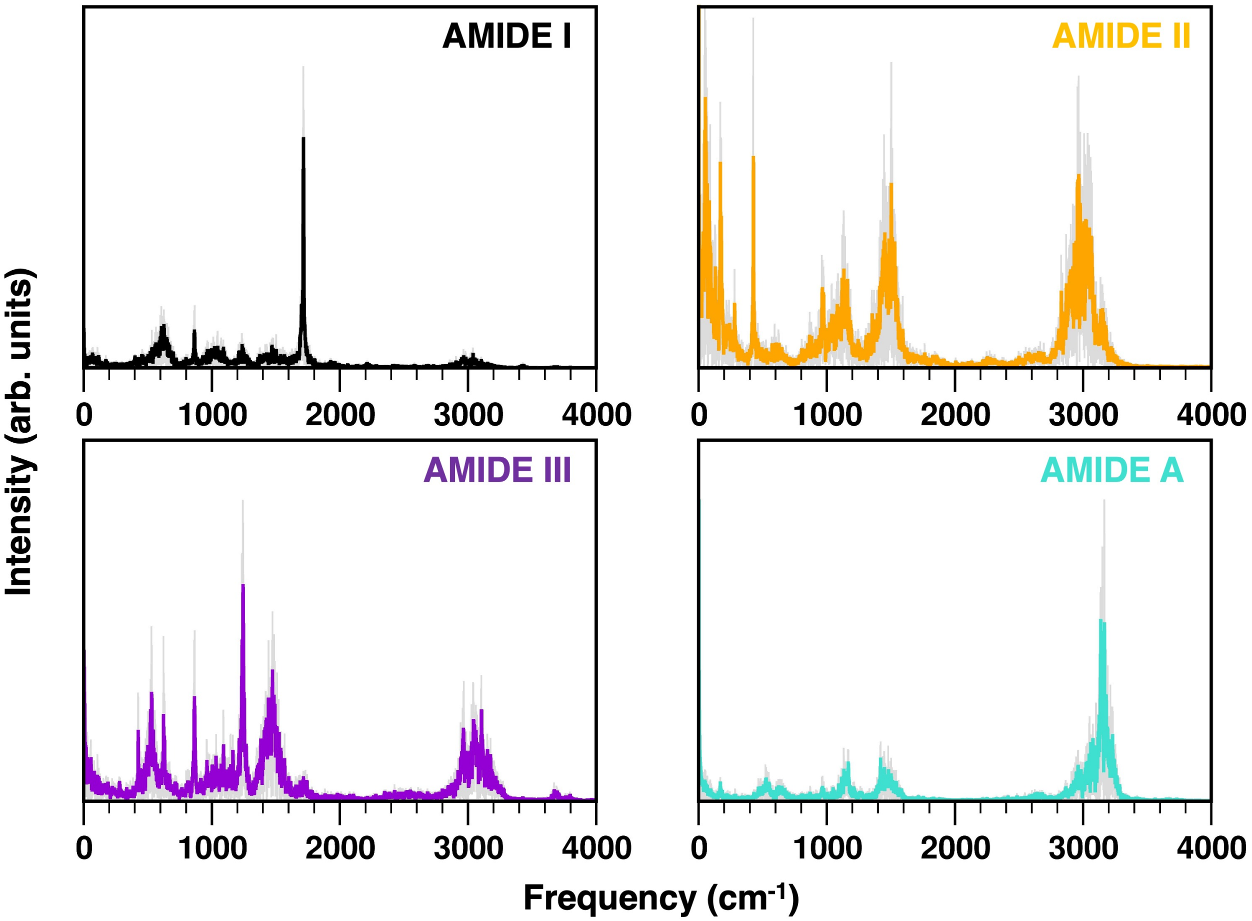

Figure S9. Vibrational spectra obtained from the Fourier transform of velocity–velocity autocorrelation functions computed from AIMD simulations in the gas phase for the tNMA$_D$ hydrogen-bond donor monomer. Each panel highlights the spectral contribution of a specific amide band: **Amide I** in the top left, **Amide II** in the top right, **Amide III** in the bottom left, and **Amide A** in the bottom right. Intensities are normalized and reported in arbitrary units as a function of frequency (cm^{-1}).

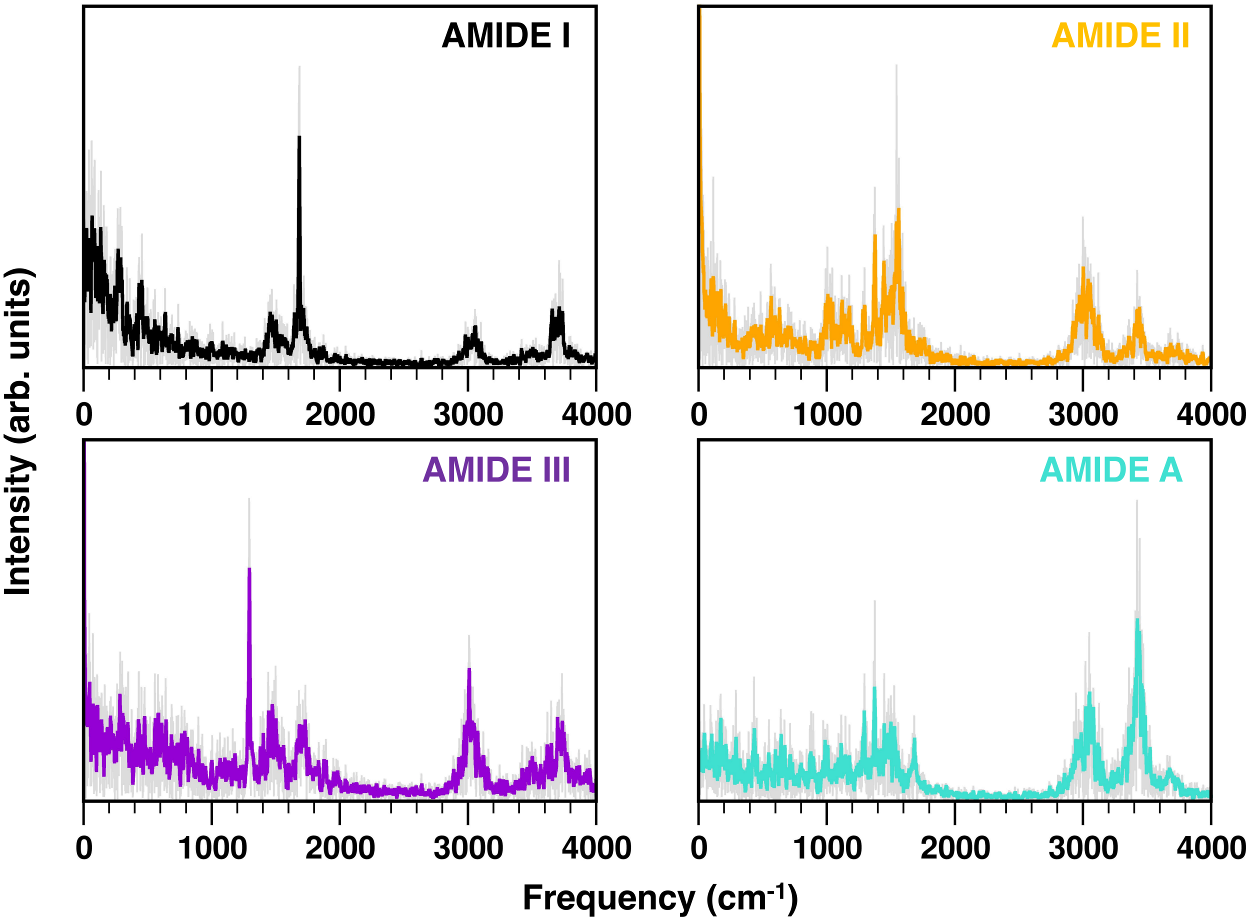

Figure S10. Vibrational spectra obtained from the Fourier transform of velocity–velocity autocorrelation functions computed from AIMD simulations in aqueous solution for the tNMA$_D$ hydrogen-bond acceptor monomer. Each panel highlights the spectral contribution of a specific amide band: **Amide I** in the top left, **Amide II** in the top right, **Amide III** in the bottom left, and **Amide A** in the bottom right. Intensities are normalized and reported in arbitrary units as a function of frequency (cm^{-1}).

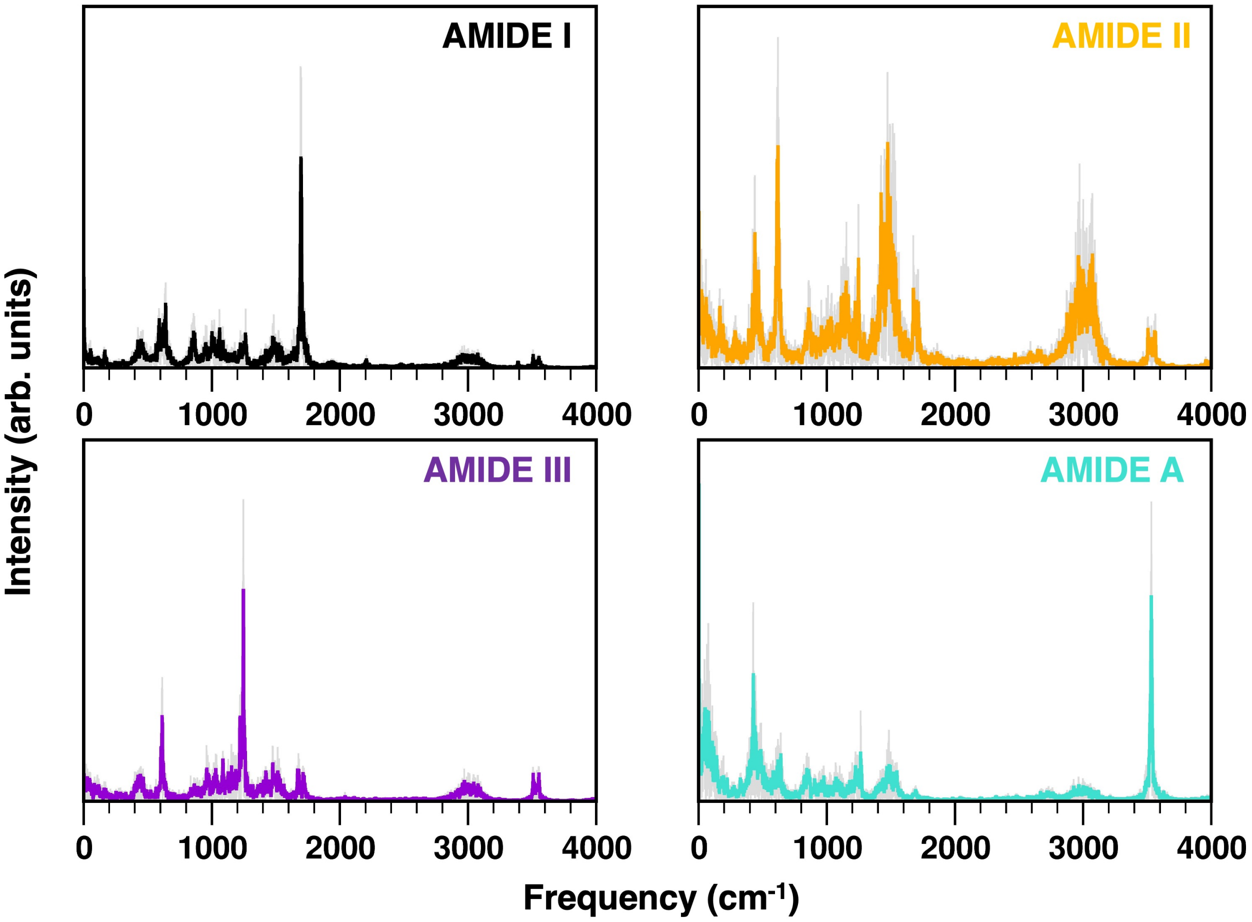

Figure S11. Vibrational spectra obtained from the Fourier transform of velocity–velocity autocorrelation functions computed from AIMD simulations in the gas phase for the $tNMA_A$ hydrogen-bond acceptor monomer. Each panel highlights the spectral contribution of a specific amide band: **Amide I** in the top left, **Amide II** in the top right, **Amide III** in the bottom left, and **Amide A** in the bottom right. Intensities are normalized and reported in arbitrary units as a function of frequency (cm^{-1}).

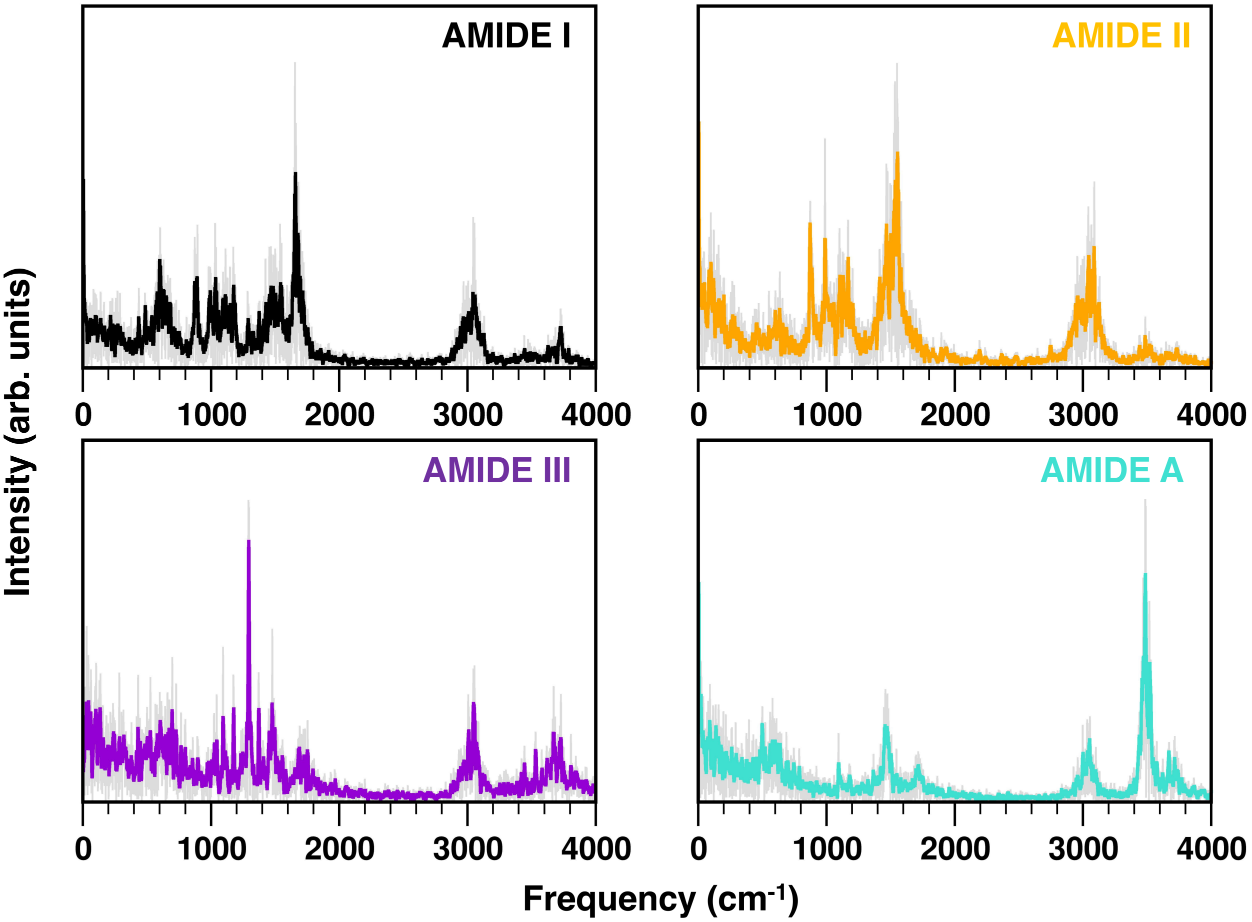

Figure S12. Vibrational spectra obtained from the Fourier transform of velocity–velocity autocorrelation functions computed from AIMD simulations in aqueous solution for the tNMA$_A$ hydrogen-bond acceptor monomer. Each panel highlights the spectral contribution of a specific amide band: **Amide I** in the top left, **Amide II** in the top right, **Amide III** in the bottom left, and **Amide A** in the bottom right. Intensities are normalized and reported in arbitrary units as a function of frequency (cm^{-1}).

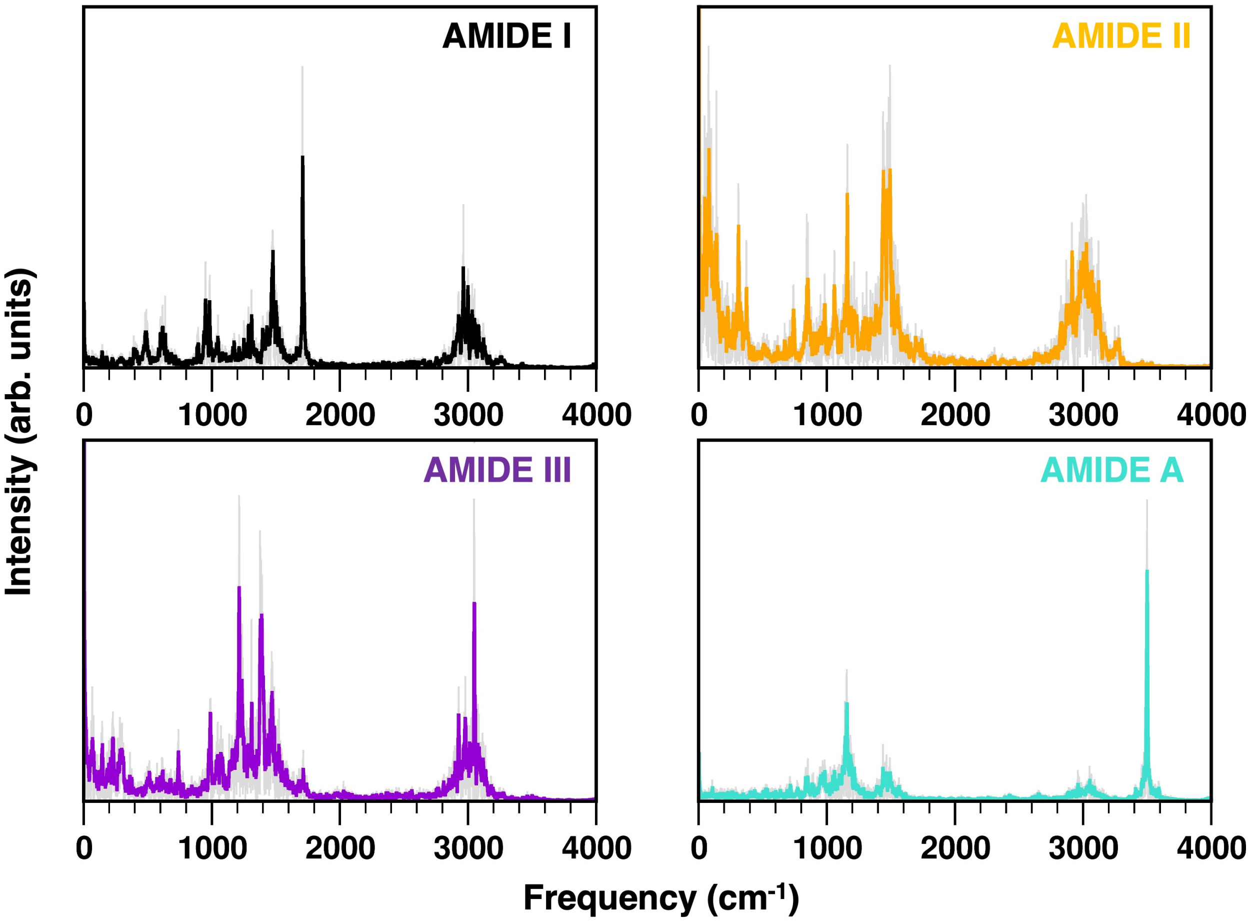

Figure S13. Vibrational spectra obtained from the Fourier transform of velocity–velocity autocorrelation functions computed from AIMD simulations in the gas phase for the AcAlaOMe system. Each panel highlights the spectral contribution of a specific amide band: **Amide I** in the top left, **Amide II** in the top right, **Amide III** in the bottom left, and **Amide A** in the bottom right. Intensities are normalized and reported in arbitrary units as a function of frequency (cm^{-1}).

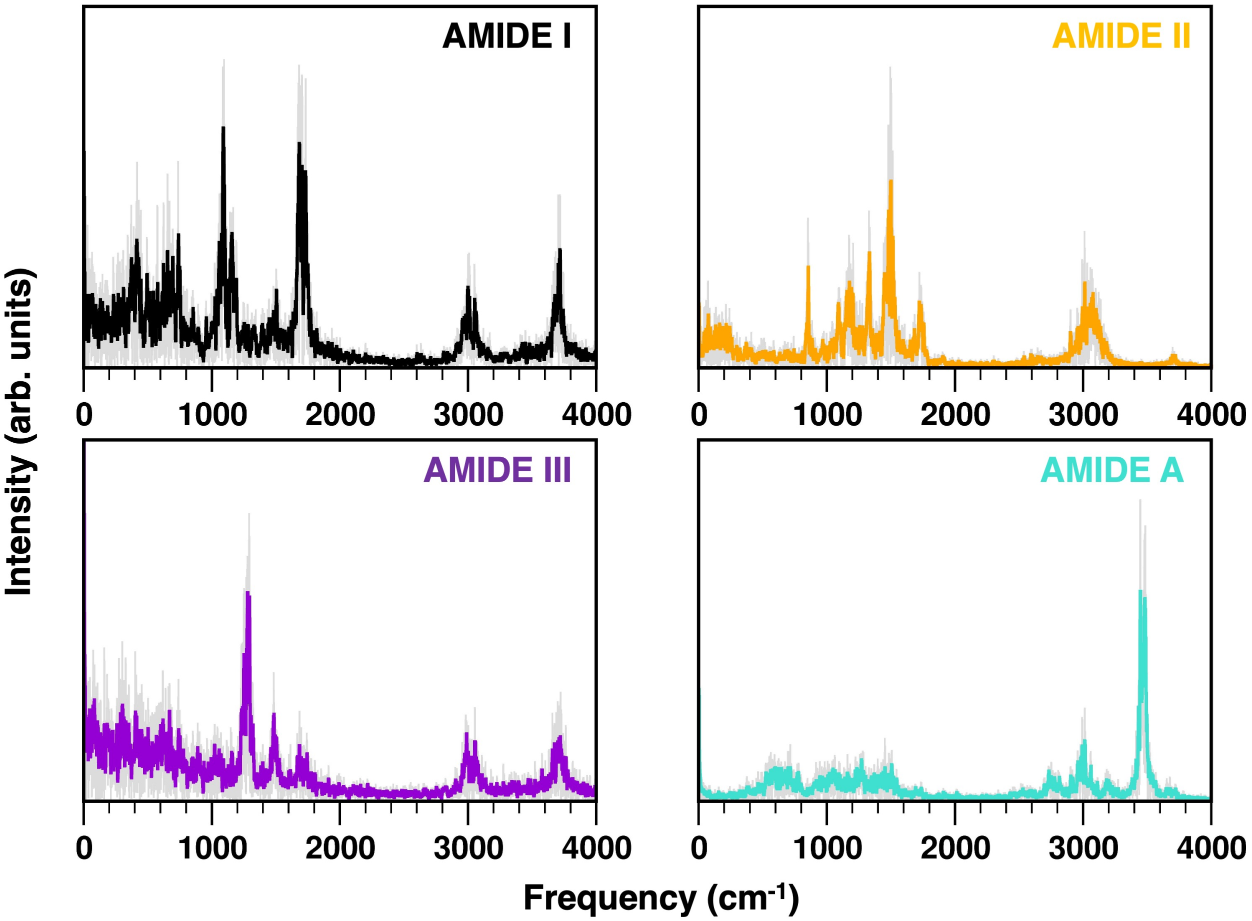

Figure S14. Vibrational spectra obtained from the Fourier transform of velocity–velocity autocorrelation functions computed from AIMD simulations in aqueous solution for AcAlaOMe system. Each panel highlights the spectral contribution of a specific amide band: **Amide I** in the top left, **Amide II** in the top right, **Amide III** in the bottom left, and **Amide A** in the bottom right. Intensities are normalized and reported in arbitrary units as a function of frequency (cm^{-1}).

REFERENCES

[1] L. Yao, B. Vogeli, J. Ying, and A. Bax, "NMR determination of amide N-H equilibrium bond length from concerted dipolar coupling measurements," Journal of the American Chemical Society **130**, 16518–16520 (2008).
